# Spatial preferences account for inter-animal variability during the continual learning of a dynamic cognitive task

**DOI:** 10.1016/j.celrep.2022.110708

**Published:** 2022-04-19

**Authors:** David B. Kastner, Eric A. Miller, Zhuonan Yang, Demetris K. Roumis, Daniel F. Liu, Loren M. Frank, Peter Dayan

**Affiliations:** 1 Department of Psychiatry and Behavioral Sciences, University of California, San Francisco, San Francisco, CA 94143, USA; 2 Kavli Institute for Fundamental Neuroscience and Department of Physiology, University of California, San Francisco, San Francisco, CA 94158, USA; 3 Howard Hughes Medical Institute, 4000 Jones Bridge Road, Chevy Chase, MD 20815, USA; 4 Max Planck Institute for Biological Cybernetics, 72076 Tübingen, Germany; 5 University of Tubingen, 72074 Tübingen, Germany; 6 These authors contributed equally; 7 Lead contact

## Abstract

Understanding the complexities of behavior is necessary to interpret neurophysiological data and establish animal models of neuropsychiatric disease. This understanding requires knowledge of the underlying information-processing structure—something often hidden from direct observation. Commonly, one assumes that behavior is solely governed by the experimenter-controlled rules that determine tasks. For example, differences in tasks that require memory of past actions are often interpreted as exclusively resulting from differences in memory. However, such assumptions are seldom tested. Here, we provide a comprehensive examination of multiple processes that contribute to behavior in a prevalent experimental paradigm. Using a combination of behavioral automation, hypothesis-driven trial design, and reinforcement learning modeling, we show that rats learn a spatial alternation task consistent with their drawing upon spatial preferences in addition to memory. Our approach also distinguishes learning based on established preferences from generalization of task structure, providing further insights into learning dynamics.

## In brief

Spatial alternation behaviors are commonly used to measure memory. Kastner et al. use experimental and computational approaches to show that rats learn spatial alternation in a manner consistent with their utilizing multiple computational features in addition to just memory and that variation in use of these features underlies inter-animal variability.

## Graphical Abstract

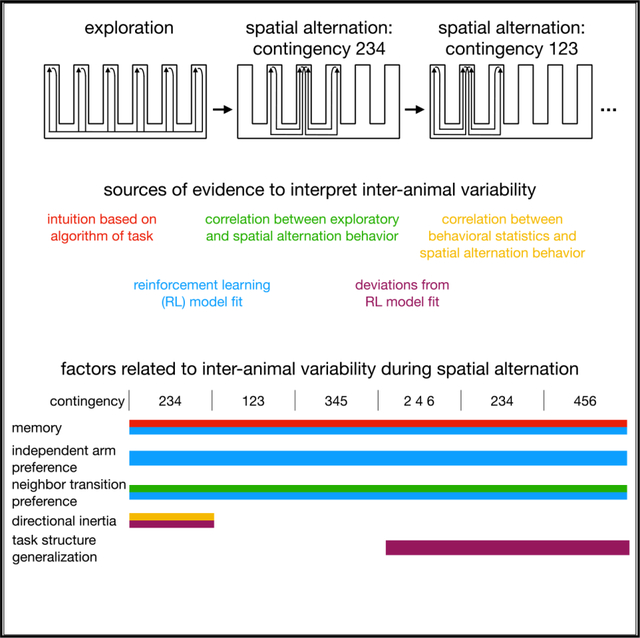

## INTRODUCTION

It is only by describing behavior accurately that we will be able to define the underlying neural computations and also understand the causal processes that lead to neuropsychiatric disease (Anderson and Perona, 2014; Krakauer et al., 2017). Animal behavior is measured through observing actions, which depend on the information the animal has about the environment, as well as the way in which it makes decisions using that information. Unfortunately, from the perspective of the experimenter, the knowledge of the animal and its decision-making processes are not directly observable.

Since these latent causes of behavior are not directly observable, it is common to hypothesize a direct link between the rules of the tasks used to evoke the behavior and the mechanisms used by the animal to learn and perform the behavior. For example, learning and memory tasks are designed to require memories for past actions. As a result, the behavior is typically interpreted in the context of memory processes, and differences in behavior between animals are interpreted as resulting from differences in memory between animals (Awasthi et al., 2019; Faraco et al., 2019; Fernandez-Ruiz et al., 2019; Jadhav et al., 2012; Mukai et al., 2019; Ribeiro et al., 2019; Ruediger et al., 2011; Shin et al., 2019; Sigurdsson et al., 2010; Vasek et al., 2016; Wang et al., 2020).

We have recently shown that this assumption does not account for the way rats learn even a simple spatial alternation task (Kastner et al., 2020). We hypothesized that rats could also be utilizing preferences that govern the way in which they interact with space, such as through preferring certain arms over others and through preferring certain transitions between arms over others (spatial preferences). However, previously used paradigms make it difficult to rigorously test that hypothesis. In the conventional way of assessing spatial alternation behavior, there is a conflation of learning the rules of the task with learning the spatial layout of the environment, since animals are exposed to the track and the rules of the task simultaneously. Therefore, it is not possible to derive an independent measurement of the presence of spatial preferences. In addition, our previous paradigm only provides a single spatial alternation contingency and thus a rather limited set of constraints on models of how such contingencies are learned. Finally, previous approaches do not limit the variability associated with animal and experimenter interaction, which could introduce additional differences (Sorge et al., 2014).

We therefore developed an experimental paradigm to determine whether incorporating spatial preferences provides predictive power to describe learning for both individual rats and groups of animals. Our paradigm combines high-throughput behavioral automation, hypothesis-driven behavioral design, and reinforcement learning (RL) modeling. We add an initial exploratory period to measure the intrinsic spatial preferences of the rats and to separate the learning of the spatial structure of the environment from the learning of the rules of the task itself. We utilize multiple spatial alternation contingencies, providing more substantial constraints on models. And finally, we use RL modeling to test explicitly various potential contributions to the learning.

We find that differences in spatial preferences can account for inter-animal variability in learning the task. Through modeling the behavior, we also gain insight into when the behavior is consistent with just utilizing memory and dynamic spatial preferences. Such learning contrasts with the case when more complex factors come into play, such as generalization about task structure. We do find evidence for the latter; however, our analysis strongly suggests that this happens later than might otherwise have been concluded.

## RESULTS

### Automated system for rats to learn a series of spatial alternation contingencies

**Figure 1.**
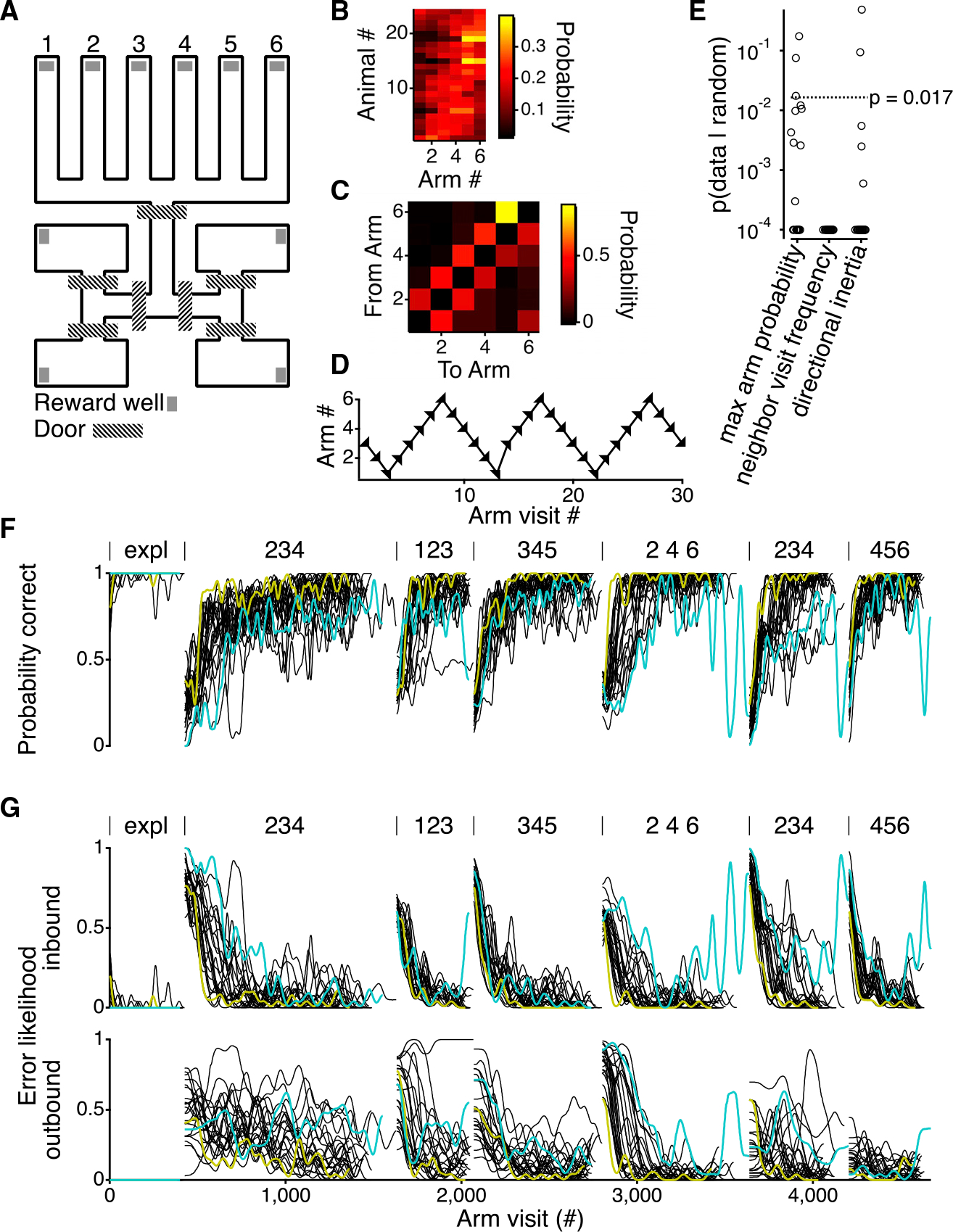
Automated behavior system for analysis of continuous spatial alternation behavior (A) Layout of automated behavior system. A six-arm track is connected to four rest boxes, each of which contains one rat during the behavior. The rest boxes are separated from the track by computer-controlled doors. (B) Arm preferences of all rats (n = 24) during the exploratory period of the behavior, where a rat can get rewarded at any arm of the track. Rats are ordered by their maximum arm number preference. (C) Example transition matrix during the exploratory period of a single rat showing the probability of going to any of the six arms when starting from each of the six arms. (D) Example arm choices (arrowheads) of a single rat during a session of the exploratory behavior. (E) Probability of seeing the maximal arm preference (left) neighbor visit frequency (middle) or directional inertia (right) given random choices between the six arms. Horizontal line shows a probability of 0.01667 (0.05 with Bonferroni correction for the three tests). As the p value was determined using 10,000 draws from distributions, the minimal value is 10^−4^. (F) Probability of getting a reward for all 24 rats. Within each contingency, curves are smoothed with a Gaussian filter with a standard deviation of 10 arm visits. Two different rats are shown in colors (yellow and teal) to indicate consistency of performance in those rats across the different contingencies. The beginning of each contingency is demarcated by vertical lines above the plot. Contingencies are indicated by the three arms that have the potential to be rewarded. (G) Error likelihoods for inbound and outbound trials for all 24 animals. Values are smoothed with a Gaussian filter with a standard deviation of 10 inbound or outbound trials and then interpolated to reflect total arm visits. Colors indicate the same rats as in (F). Contingencies are indicated as in (F).

Our goal is to understand the computations that underlie spatial alternation behavior. To do this, we sought to measure and then model the entire course of learning. To standardize the behavior and reduce potential effects of experimenter-subject interactions on learning (Sorge et al., 2014), we developed an automated behavioral system (Brunton et al., 2013; Poddar et al., 2013; Rivalan et al., 2017) that requires minimal animal handling. This system also enables the measurement of behavior across many animals throughout the entire course of learning and performance of the task (Figure 1A; see STAR Methods).

Our previous work suggested that accurate descriptions of learning might require dynamic preferences, defined as tendencies that change in response to reward for animals to prefer specific locations or specific transitions between locations (Kastner et al., 2020). It was therefore critical to measure the initial values for these preferences. Furthermore, we sought to disambiguate the learning of the task from the learning of the space of the task. Therefore, prior to the rats beginning the spatial alternations task, they had multiple sessions of exploration on the track (see STAR Methods).

These exploration sessions revealed multiple preferences. First, individual rats showed preferences towards visiting specific arms (Figures 1B and 1E). Twenty-one of the twenty-four rats showed significant deviation from a random arm visit pattern (p = 1.1 × 10^−8^; see STAR Methods). Second, rats also had a large propensity to transition from their current arm to neighboring arms (Figures 1C and 1E), with all 24 rats showing significant deviation from randomly transitioning between arms, even given their individual arm visit probabilities (p = 6.3 × 10^−8^; see STAR Methods). And finally, the rats exhibited directional inertia, whereby they continue to go in the same direction. Directional inertia is calculated as the frequency of an animal going in the same direction as it did on the immediately preceding trial (Figures 1D and 1E). A partially different 22 out of 24 rats showed significant deviation from random directional inertia, even accounting for their individual transition probabilities (p = 1.7 × 10^−6^; see STAR Methods).

Given the existence of these preferences, we asked whether those preferences play a role in learning. Following this initial exploratory period, and without any external signal to indicate a change, rats were sequentially exposed to different spatial alternation contingencies. The six arms of the track allow for the learning of multiple spatial alternation contingencies (Singer and Frank, 2009; Singer et al., 2010). The animals had to learn six different contingencies (Figure 1F). These contingencies help constrain the models for each individual animal and enable cross-validation across the contingencies.

In each contingency, only three arms had the potential to deliver reward. Reward is delivered within a contingency if the rat alternates between the outer arms after every visit to the center arm. For instance, if the contingency was at arms 234, to get reward, the rat would have to follow the sequence 3–4-3–2-3–4-3, etc. The rats get rewarded for any correct arm visit where they broke the infrared beam at the reward well, so in the previous example, the rat would receive a total of seven rewards. Following previous studies in a related environment (Jadhav et al., 2012; Kim and Frank, 2009), we defined inbound trials as trials where the rat starts from an arm that is not the center arm (arm 3 in this example) and outbound trials as trials where the animal starts at the center arm of the contingency.

Performance improved on each of the contingencies, such that, by the end of each, rats typically made few outbound or inbound errors (Figures 1G and S1B). There was, however, substantial and systematic variability across animals, where individual animals consistently showed higher or lower performance across contingencies (see yellow and cyan colored lines in Figures 1F and 1G for examples). This variability provided an additional goal for our modeling, in that an ideal model would capture not only the overall learning of the group but also the differences among individuals.

### Modeling framework

We note that our goal was not to perfectly recapitulate all aspects of each animal’s behavior, as such a goal is well beyond our current understanding. Instead, we sought to develop a simple, interpretable model that could capture learning rates across at least a subset of contingencies. Such a model would allow us to determine whether incorporating spatial preferences was important for describing behavior. That model, if it could be fit to individual animals, could also help us quantify differences in behavior among individuals. Finally, areas of lack of fit would provide a clear direction forward for future augmentation.

We use a similar modeling framework as our previous study (Kastner et al., 2020). For clarity, we describe and motivate the choices of that modeling framework. The framework uses a simple algorithm that, like the animals, does not require acausal information, can alter its internal information based upon its choices and rewards to increase the expected return of reward, and can work in the face of partial observability. This led us to the actor-critic class of RL accounts trained by the REINFORCE policy gradient algorithm (Williams, 1992) and employing a form of working memory (Hochreiter and Schmidhuber, 1997; Todd et al., 2009). Variants of REINFORCE are popular choices for characterizing animal learning behavior in RL paradigms (Suri and Schultz, 1999), and there is also evidence of its utility in modeling human behavior (Li and Daw, 2011).

**Figure 2.**
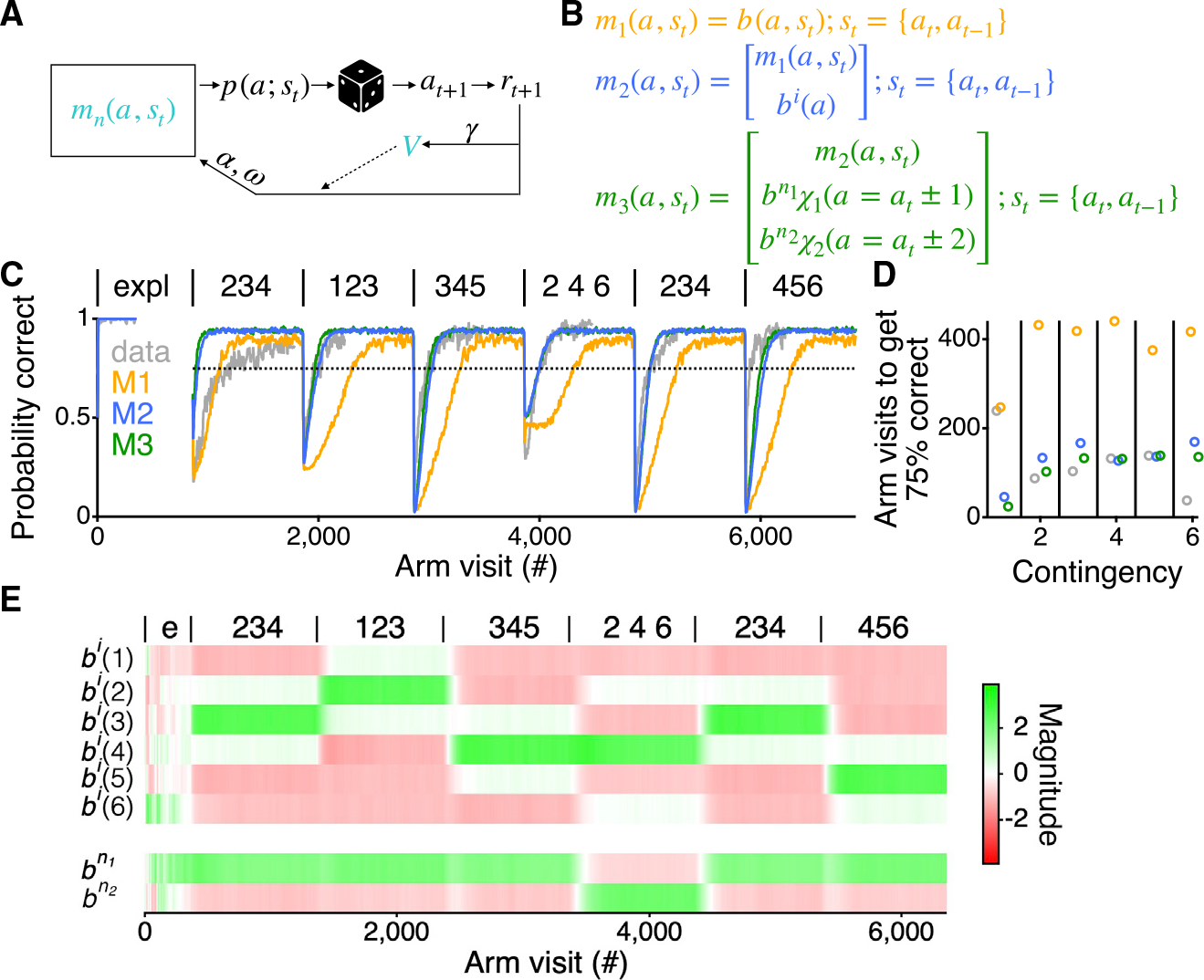
RL model with working memory and dynamic preferences can learn as rapidly as the rats (A) Diagram of RL agent. Colored symbols, *m*_*n*_(*a*,*s*_*t*_) and *V*, indicate the components that change as the agent goes to arms, *a*, and does or does not get reward, *r*. (B) The components of the propensities, *m*_*n*_(*a*,*s*_*t*_), for the different models. The state of the agent, and therefore the probability of transitioning to each of the arms, *p*(*a*;*s*_*t*_), is defined by the current arm location, *a*_*t*_, and the previous arm location,*a*_*t*−1_, of the agent. *b*^*i*^(*a*) is the independent arm preference. $b^{n_{1}}\chi_{1}\left(a=a_{t}\pm 1\right)$ and $b^{n_{2}}\chi_{2}\left(a=a_{t}\pm 2\right)$ are the preferences to transition to a neighbor one or two arms away, respectively. (C) Average reward probability of all animals (n = 24) across all contingencies (gray) and average behavior of 200 repeats of the models with parameters chosen to maximize the rewards received across all contingencies. The models were given extra arm visits to reach asymptotic behavior. Dotted horizontal line shows 75% probability correct. Contingencies are indicated as in Figure 1F. (D) Number of trials to pass 75% probability correct for the data (gray) and models. Colors refer to the different models from the previous panels. (E) The average individual arm preferences (*b*^*a*^) and neighbor arm preferences (*b*^*n*1^ and *b*^*n*2^) across all contingencies and repeats of M3 for the parameters that maximize the reward shown in (C) (green). The values shown are those prior to passing through the exponential for the softmax. Contingencies are indicated as in Figure 1F, with exploration demarcated as “e.”

That algorithm allows us to specify a family of models with a common form. The models describe the behavior of an agent choosing an arm on trial *t*, which we write as *a*_*t*_. The choice of *a*_*t*_ depends probabilistically on an internal characterization of its state, s_*t*−*1*_, which can contain information about past arm choices. This dependence arises through a collection of action preferences or propensities *m*(*a*,*s*_*t*_), such that actions with higher propensities are more likely to be chosen. The propensities are updated as a function of reward. The full details of the equations involved are provided in the STAR Methods. In brief, a conventional softmax function converts the propensities to probabilities, *p*(*a*;*s*_*t*_), of choosing to go to arm *a*_*t+1*_= *a* on this trial (Equation 1). Via the rules of the task, this choice of arm then determines whether the model receives a reward, *r*_*t*+*1*_, and causes the state to update to *s*_*t*+1_. This reward is used to calculate the prediction error, *δ*_*t*_, using the value function of the critic at states *s*_*t*_ and *s*_*t*+1_, *V*(*s*_*t*_) and *V*(*s*_*t*+1_) (Equation 2). *δ*_*t*_ is then used to update *V*(*s*_*t*_) (Equation 6) and the factors governing the propensities *m*(*a*,*s*_*t*_) (Equations 3, 4, and 5). Finally, new propensities *m*(*a*,*s*_*t*+1_) are calculated, at which point the process repeats with the agent choosing its next arm to visit (Figures 2A and 2B).

All the models described below have three parameters, each of which takes values between 0 and 1: (1) the temporal discount factor, γ, determines the weighting of rewards in the farther future in defining the long-run values of states (and thus in calculating the prediction error, δ; Equation 2); (2) the learning rate, α, determines how much δ updates the propensities and the value function (Equations 3, 4, 5, and 6); and (3) the forgetting rate, ω, determines how quickly the propensities and the value function decay towards 0 (Equations 3, 4, 5, and 6), at which point there would be no preference for visiting any particular arm in any state. ω enables the model to encompass the nonstationarity of the task by constantly depreciating old information.

The framework described above falls into the category of model-free (MF) RL agents, which typically learn slower than animals. Therefore, to develop a model that has the potential to learn as quickly as individual rats, we started by comparing the best a model could do to the average behavior of the rats (Figures 2C and 2D). This provided a straightforward way to determine whether the model had the potential to fit individual animals because, if the best version of the model could not learn as quickly as the animals, there would be no chance for it to capture the learning of individual animals.

### Memory alone is not sufficient

We previously demonstrated that a model with “working” memory alone does not capture the way rats learn a simple spatial alternation task (Kastner et al., 2020). We replicate and extend that finding in this more complex environment using our first model (M1). As in the previous work (Kastner et al., 2020), we added a memory component following an approach by Todd et al. (2009), where the state of the model is augmented by a memory unit that stores the immediate past action. This enables the model to make decisions based upon current and past information. Such a strategy has been used to learn common rat behavioral tasks (Zilli and Hasselmo, 2008) and exhibits features of rat behavior (Lloyd et al., 2012). In all the models, the state, *s*_*t*_ = {*a*_*t*_,*a*_*t*−*1*_}, includes both the current and the most recent past arm (Figure 2B). For model M1, the propensities are *m*_1_ (*a*, *s*_*t*_) = *b*(*a*|*a*_*t*_, *a*_*t*−1_). For each state, *b*(*a*|*a*_*t*_, *a*_*t*−1_) contains five numbers governing the propensity to make a transition from the current arm to each of the other five arms. Returning to the same arm is not allowed in the model, as it was never rewarded in the behavior.

This working memory RL agent has perfect memory of the immediate past and has the capacity to perform each contingency well; however, it learns to do so far slower than the average of the rats (Figures 2C and 2D), even when the parameters are set to maximize the obtained reward. With M1, good performance on the first contingency arises at the correct timescale—something that will be discussed further below—but performance on all the subsequent contingencies improves much slower than the rats. For contingencies 2–5, M1 reached 75% correct 2.7–4.9 times slower than the average performance of the rats, and for contingency 6, M1 was 10.9 times slower.

### Arm and transition preferences, combined with memory, enable the model to learn as rapidly as the rats

Given the failure of M1 to show relevant learning rates, we asked whether the incorporation of dynamic preferences would be sufficient to enable rapid learning, as was the case for the simpler three-arm version of the task (Kastner et al., 2020). To capture the preferences, we added components to the propensities of the model. We begin by adding a single term for each arm to capture the individual arm preferences shown by the animals (Figure 1B). This yields model M2, where *m*_2_ (*a*, *s*_*t*_) = *b*(*a*|*a*_*t*_, *a*_*t*−1_) + *b*^*i*^(*a*) (Figure 2B). The term *b*^*i*^(*a*), a dynamic independent arm preference, provides the agent with additional preferences to choose specific arms next, independent of its current or past locations. As with the state-dependent propensity terms, *b*^*i*^(*a*) are updated by δ_t_ through the process of learning (Figure 2E). Importantly, adding this term allows us to capture both the fact that the animals may prefer specific arms before beginning the learning of the alternation contingencies and that these preferences can be dynamic and shaped by reward. Importantly, including this term or any other preference-related term does not entail adding any additional free parameters to the model.

Including the dynamic independent arm preference yields an agent that can learn much more quickly but still failed to match the learning rates of the rats, even when using the parameters that maximized the reward M2 could receive (Figures 2C and 2D). M2 learned the first contingency faster than the animals, reaching 75% correct five times faster than the rats. By contrast, for contingencies 2–5, M2 reached 75% correct 1.0–1.6 times slower than the average performance of the rats, and for contingency 6, M2 was 4.4 times slower (Figure 2D).

The failure to match learning rates led us to incorporate an additional preference observed in the animals, a dynamic transition preference. This yields model M3, for which $m_{3}\left(a,\;s_{t}\right)\;=\;b\left(a|a_{t}\;,\;a_{t-1}\right)+b^{i}(a)\;+\;b^{n_{1}}\chi_{1}\left(a\;=\;a_{t}\;\pm \;1\right)\;+\;b^{n_{2}}\chi_{2}\left(a\;=\;a_{t}\pm \;2\right)$, where χ_n_ () is the characteristic function that takes the value of 1 if its argument is true (Figures 2B and 2E). The additional propensity components capture the preference of the animals to transition to neighboring arms that are either one, *b*^*n1*^, or two arms, *b*^*n2*^, away, independent of the current location of the animal (Figure 1C). The term to capture transitions two arms away, *b*^*n2*^, was included to provide the model with the potential to capture the 4^th^ contingency (2 4 6), where it is necessary to skip arms to receive reward. The combination of the two terms allows the model a flexible metric for the spatial organization of the arms—something that is missing in M1 and M2. Beyond just being convenient for modeling the task, we found this propensity in the behavior of the animals as well. During the exploratory period, if the animals do not go to a neighboring arm, they go two arms away 53.5% ± 2.9% of the time, a value far greater than would be expected by chance, even when controlling for the arm visit probability of each animal (p = 0.004). With model M3, as with the previous models, these state values update using the same three parameters.

M3, using the parameters that maximized reward, more closely approximates the behavior of the rats (Figures 2C and 2D). While M3 reached 75% correct 10 times faster than the rats on the first contingency and 3.6 times slower for contingency 6, for contingencies 2–5, M3 reached 75% correct at rates more similar to the average performance of the rats when compared with M2 (p < 1 × 10^−14^; see STAR Methods).

Both the dynamic independent arm preference and dynamic transition preferences are necessary for the rapid learning rate. A model that contains the state-based transition preference and dynamic transition preference, $m\left(a,\;s_{t}\right)=b\left(a|a_{t},\;a_{t-1}\right)+b^{n_{1}}\chi_{1}\left(a=\;\;a_{t}\pm 1\right)+b^{n_{2}}\chi_{2}\left(a=\;\;a_{t}\pm 2\right)$, but does not contain the independent arm preferences learns too slowly. This model, at best, learns most contingencies over two times slower than the rats (Figure S2).

### Model with memory and arm and transition preferences fits individual animals

M3, despite its relative simplicity, matched the average learning rates of the animals for some contingencies. This in turn suggested that it could capture important aspects of the behavior of individual rats. For the fit to an individual rat, we forced the model to make the same sequence of arm visits as the animal during the initial exploratory phase, effectively using the data of the animal to inform the initial condition of the model. We then fit aspects of selected contingencies, testing how well the resulting parameters predicted the behavior in the other contingencies.

To determine the best fitting parameters, we used an approximate Bayesian computation (ABC) method (Lintusaari et al., 2017), consistent with other studies using RL agents, to fit rodent behavior (Lloyd et al., 2012; Luksys et al., 2009). ABC methods find parameters such that the average behavior of the model when operating in the task, choosing stochastically, matches as well as possible that of an individual animal, according to some suitably chosen statistics. We averaged 200 repeats of the model and chose as statistics the inbound and outbound performance for the contingencies we fit. We then evaluated the fit of the model to each animal by calculating the root mean square (RMS) difference between the model and data on inbound and outbound trials.

We found that, even though the model was able to fit to the inbound and outbound errors of the first contingency (Figure S3A), the parameters from those fits did a poor job of capturing the behavior of the animals on subsequent contingencies (Figure S3B). This failure was not surprising, given that the first contingency was an outlier when evaluating the maximal reward the models could receive (Figure 2C). We will return to understand this difference below.

Therefore, we chose to fit the second and third contingencies. These contingencies are the most representative for this task, as both (1) follow other simple contingencies and (2) occur before the hardest, fourth, contingency, for which the required alternation involves skipping neighboring arms. To verify that the additional preferences of M3 were necessary for the fit to individual animals, we also fit to M1 and M2.

**Figure 3.**
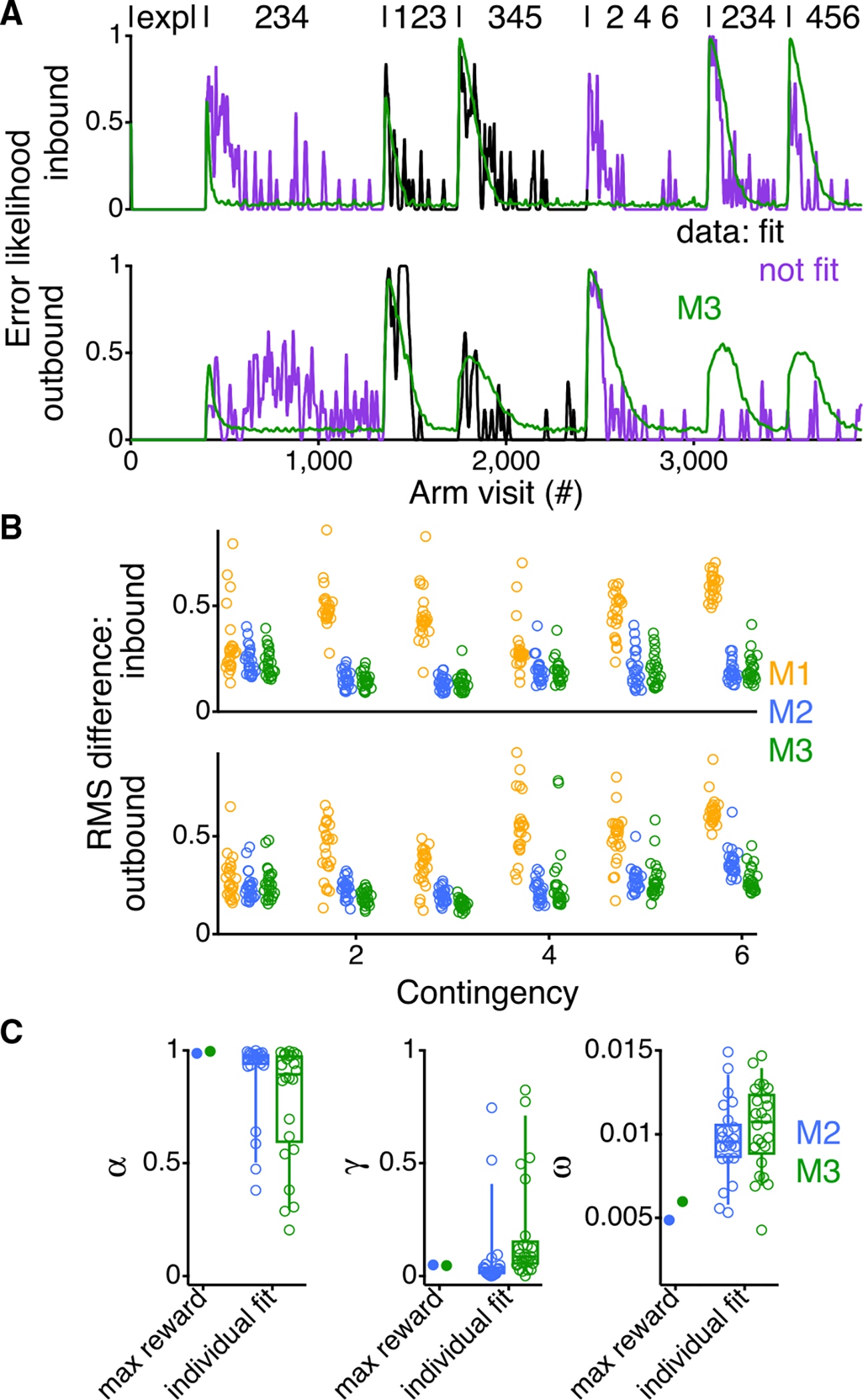
Fitting model to individual animals to capture variability between rats (A) Inbound and outbound error likelihood for an individual animal across all contingencies (purple or black). Values are smoothed with a Gaussian filter with a standard deviation of 2.25 errors and then interpolated across arm visits. In green is the average behavior of 200 repeats of the model using the parameters that minimize the RMS difference between the model and the animal during the second and third alternation contingencies (black). Purple indicates data that were not included in fitting the model. Contingencies are indicated as in Figure 1F. (B) RMS difference between the model and the data for all animals (n = 24) for the inbound and outbound errors for each contingency for the different models. (C) Comparison of the parameters for the fits of individual animals (open circles) to the parameters that maximize rewards (closed circles) from Figure 1C. Box plots show the median, interquartile range, and the range between the 9^th^ and 91^st^ percentile of the data.

The fits of the second and third contingencies (Figure 3A) confirmed that M3 fit the individual animals better than M2 and M1 (Figure 3B). Specifically, both M2 and M3 fit inbound and outbound errors with lower RMS difference as compared with M1 (p < 10^−6^; paired permutation test), and M3 improved upon M2’s performance for outbound errors (p = 1.4 × 10^−4^; paired permutation test). These findings indicate that incorporating all three observed propensities—memory, independent arm, and neighbor transition preferences—improves the fit of the model to the data. We note that there remain clear situations when M3 still does not fit the data well and return to this observation below.

### Individual model fits capture variability in behavior

When fit to the second and third alternation contingencies, M3 yielded parameters that were much more variable across animals, suggesting that it might capture individual differences during these two contingencies. When compared with M2, the M3 fits to all 24 rats had an interquartile range 7.8 times larger for α (0.39 versus 0.05; p = 0.004; paired permutation test), 3.0 times larger for γ (0.11 versus 0.04; p = 0.001; paired permutation test), and 1.8 times larger for ω (0.004 versus 0.002; p = 0.046; paired permutation test).

**Figure 4.**
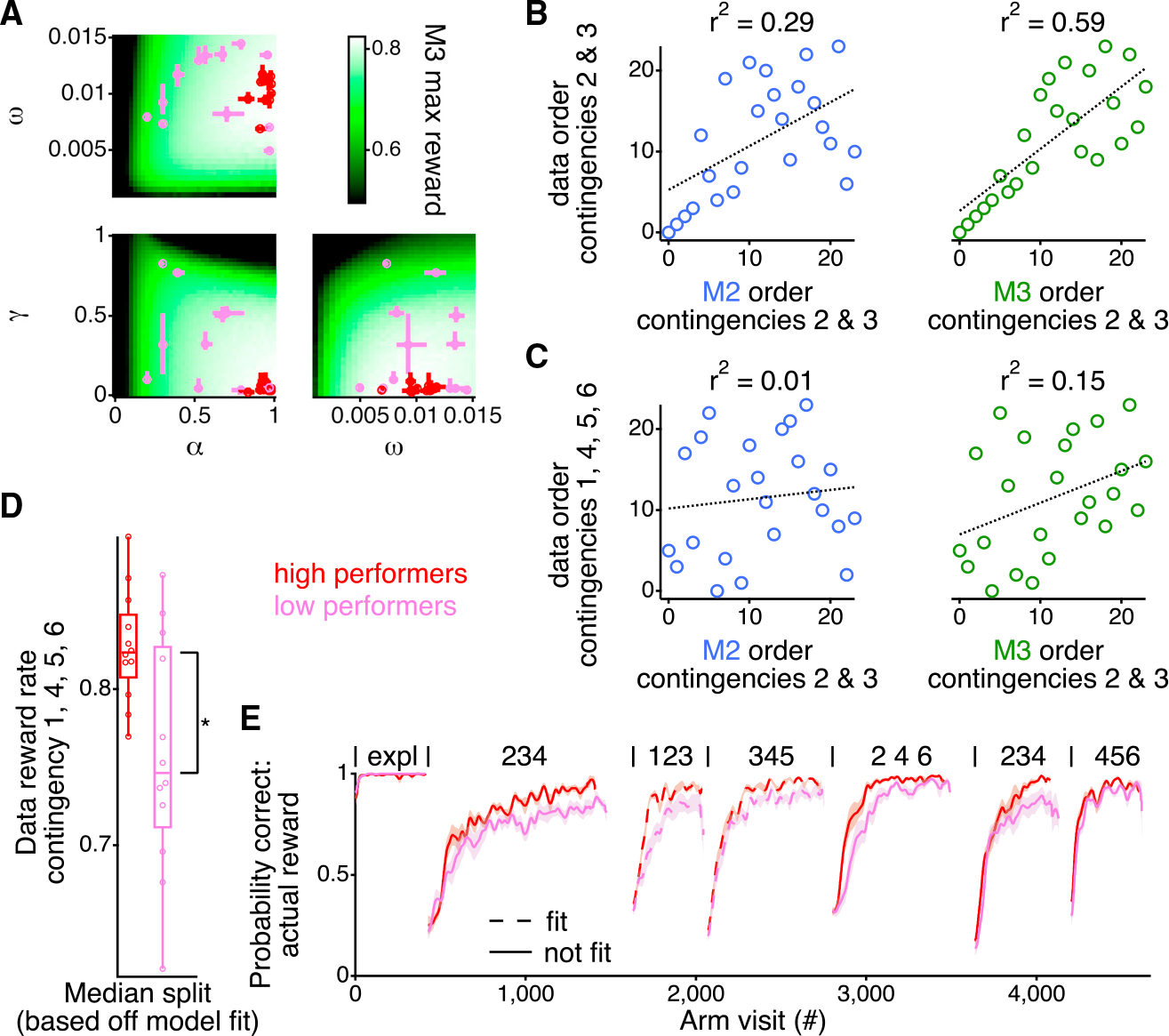
M3 captures individual variability in animal performance (A) Three-dimensional space of parameters projected down onto all pairs of parameters. The median and interquartile range of 20 fits for each animal are plotted as the red or pink dot with errors bars in both axes. Red and pink colors reflect the median split of animals as shown in (D) and (E). Color scale in background is the maximal reward rate during the second and third contingency for the pair of parameters across all values of the third parameter. (B) Ordering of the animals based on the actual reward rate during contingencies 2 and 3 as a function of the ordering of the animals based upon the model reward rate during contingencies 2 and 3, for M2 (left) and M3 (right). (C) Ordering of the animals based on the actual reward rate during contingencies 1, 4, 5, and 6 (those not fit by the model) as a function of the ordering of the animals based upon the model reward rate during contingencies 2 and 3, for M2 (left) and M3 (right). For (B) and (C), the dotted line shows a linear fit. (D) Box plots showing the data, median, interquartile range, and 9^th^–91^st^ percentile for the actual reward rate of the animals during contingencies 1, 4, 5, and 6 when split by the model reward rate during contingencies 2 and 3. *p < 0.05. (E) Average (±SEM) probability correct across all contingencies for the grouping by the median split of the M2 reward rate for contingencies 2 and 3. Contingencies are indicated as in Figure 1F. Solid lines indicate contingencies that were not fit by the model, and dotted lines indicate those contingencies that were fit by the model.

Individual animals achieved different reward rates during the second and third alternation contingencies (Figure 1F). Along with capturing the overall structure of the learning of all animals, it is important for a model to match these differences—something achieved, to a large part, by model M3. First, M3 has available a broad landscape of reward rates according to different settings of its parameters, and the parameters for the fits to individual animals use that broad range (Figure 4A). Second, we compared the fit of M2 and M3 with the variability in reward rates. M3 does a better job than M2 for accounting for the contingences for which the model was (Figure 4B) and was not (Figure 4C) fit. For the former, we ordered the animals based upon the actual reward rate the animals received during the second and third alternation contingencies and compared that with the order of the animals based on the reward rate the model received on the second and third alternation contingencies when fit with either M2 or M3. M3 captured 58.8% of the variance in the ordering of reward rates of the animals during contingencies 2 and 3, which was substantially larger than the 29.1% captured by M2 (p = 0.017; paired permutation test). Thus, M3 better captured the relative performance of the animals on these contingencies.

That strong correlation is a necessary, but not sufficient, condition for M3 being considered a good model. A good model should also make accurate predictions on new data. We next sought to determine whether M3 also did a better job of predicting the ordering of the reward rates on contingencies to which it was not fit (Figure 4C). We found that the ordering of the model reward rates from contingencies 2 and 3 captured 15.3% of the variance in the performance of the animals to the contingencies that were not fit by the model (1, 4, 5, and 6), which is substantially larger than the 1.3% captured by M2 (p = 0.017; paired permutation test). Importantly, M3 captured the same amount of variance as the actual reward rates of the animals in contingencies 2 and 3 (*r*^*2*^ = 10.6%; p = 0.03; paired permutation test). That indicates that M3 does at least as good a job of predicting the reward rate of the animals as the reward rate of the animals themselves.

An examination of the reward rates confirmed these conclusions. We performed a median split based on the reward rate of the model to contingencies 2 and 3. The higher performing half of the rats showed a significantly greater overall reward rate on the remaining contingencies (1, 4, 5, and 6) compared with the lower performing half of the animals (Figure 4D; p = 0.02; rank sum test). The average performance of the higher performing half of the rats was consistently larger than the lower performing half across all the contingencies, even though the median split was made off the reward of the model for contingencies 2 and 3 (Figure 4E). The higher performing rats had a reward rate 10.0% larger during the first contingency, 9.7% larger during the fourth contingency, 9.6% larger during the fifth contingency, and 4.8% larger during the sixth contingency.

### Model agnostic analysis confirms importance of neighbor preference

The modeling provides strong support for the importance of the dynamic preferences for the rapid learning of this spatial alternation task. Adding the neighbor arm preference was critical for capturing the individual variability among rats in learning this task (Figures 3C and 4). That observation led us to ask whether the neighbor bias could also account for other aspects of behavioral performance.

**Figure 5.**
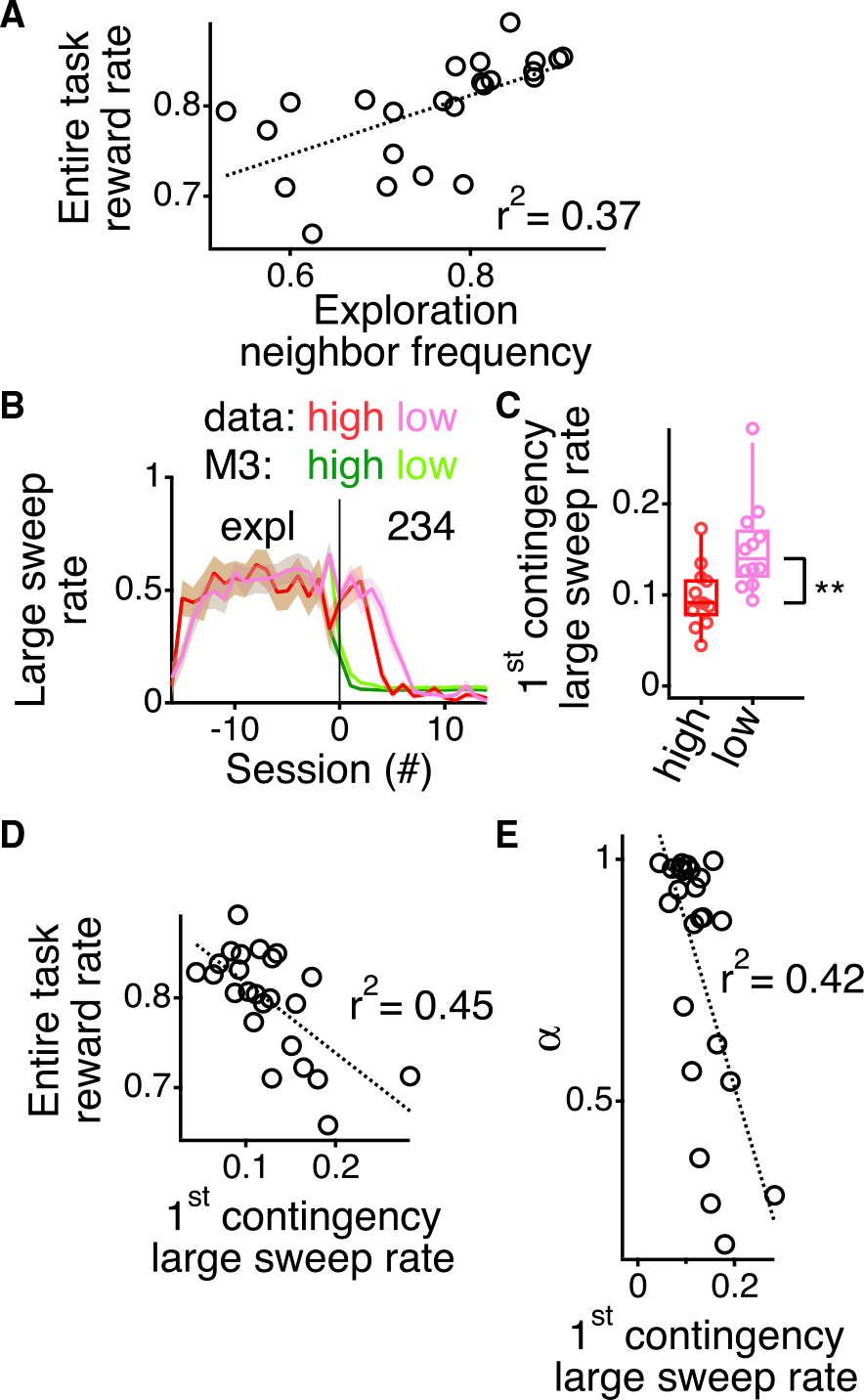
Spatial preferences account for variability in reward rate across animals (A) Average reward rate across all six alternation contingencies plotted relative to average neighbor transition frequency during the exploratory period for each animal. (B) Average (±SD) large sweep rate (greater than three arms) for each session during the exploratory period and first alternation contingency. Session 0 is the first session of the first alternation contingency. Animals are split into high (red) and low (pink) performers based upon median split from Figure 4. The same measurement is calculated off the 200 repeats of the model using the fitting parameters for each of the animas. M3 split is based upon the same grouping as the animals. The solid vertical line demarcates the transition between exploration and the first alternation contingency. (C) Box plot showing the data, median, interquartile range, and 9^th^–91^st^ percentile of the large sweep rates across the entire first contingency for the high- and low-performing animals. **p < 0.005. (D) The average reward rate across all six alternation contingencies plotted relative to the large sweep rate during the first alternation contingency for each animal. (E) Learning rate (α) from the individual fits of model M3 to each animal (fit for second and third alternation contingencies) plotted relative to the large sweep rate during the first alternation contingency for each animal. For (A), (D), and (E), dotted line shows linear fit.

Consistent with this possibility, we found that the neighbor bias from the exploratory period of the task relates to overall performance on the alternation task. During the exploratory period, we calculated the frequency with which each rat visits the neighboring arm. There was a range of preferences across the rats for neighboring arms during the exploratory period, which correlated with the average reward rate across all contingencies for each animal (Figure 5A; p = 0.0016; *r*^*2*^ = 0.37). Thus, rats that demonstrate a stronger preference for visiting neighboring arms during exploration tend to obtain more rewards during spatial alternation.

### Additional preference governs slower learning of first alternation contingency

The ability of M3 to fit the second and third contingencies argues against the hypothesis that the rats learn the subsequent contingencies faster than the first contingency because they generalize structural information about the task by the end of the first contingency. Due to its model-free nature, M3 has no capacity to generalize information about the task. Therefore, the rats could be learning the task through utilizing memory and spatial preferences without any understanding about the structure of the task. However, that then raises the question as to why the learning of the first contingency is so slow.

It is possible that learning the first contingency of the task draws upon preferences that were not included in the model. Rats exhibit directional inertia during the exploration period (Figures 1D and 1E). M3 did not include this preference, raising the possibility that the slower learning of the first contingency could be due to the presence of directional inertia. Directional inertia leads to large sweeps across the track (Figure 1D), and sweeps larger than three arms are counterproductive for the alternation task. Thus, if the behavior of the rats during the first contingency was influenced by the presence of larger sweeps, the rats would learn the first contingency slower than the model and subsequent contingencies.

Consistent with this possibility, the rats, but not the model, show large sweeps that persist into the first contingency. We calculated the proportion of arm visits that were a part of a large sweep (greater than three arms) during the exploratory period and into the first alternation contingency (Figure 5B). The values are identical between the animals and the model during the exploratory period because we force each model to follow the same series of arm visits as the individual rats (see STAR Methods). At the transition to the first contingency, M3 drops to a low baseline level of large sweeps. In contrast, the rats persist with an elevated large sweep rate after the transition to the first alternation contingency (Figure 5B).

To provide further evidence that persistent large sweeps led to slower learning of the first contingency, we evaluated the large sweep rates of the higher and lower performing rats, as determined by the median split from the model fit to the second and third contingencies (Figure 4C). The higher performing rats dropped their large sweep rate faster than the lower performing rats (Figure 5B), with the higher performing rats having a lower overall large sweep rate in the first contingency compared with the lower performing rats (Figure 5C; p = 0.003; rank sum test). These two groups did not show any difference in large sweep rates during the exploratory period (p = 0.55; rank sum test). This is consistent with the higher performing rats more quickly learning to not perform large sweeps.

If so, then animals that learn faster should be able to overcome their preference for directional inertia more quickly. Indeed, that was the case. We calculated the large sweep rate from the first contingency, where fewer large sweeps would be expected to be associated with faster suppression of this preference. We found a strong inverse correlation between the reward rate for the entire task and the first contingency large sweep rate (Figure 5D; p = 6.0 × 10^−4^; *r*^*2*^ = 0.42). Consistent with the removal of the large sweeps being a function of the learning capacity of the animals, there was also strong inverse correlation between the learning rate, α, of the model (fit only to the second and third contingencies) and the first contingency large sweep rate (Figure 5E; p = 6.0 × 10^−4^; *r*^*2*^ = 0.42) with a also accounting for α large fraction of the variance of the overall reward rate (p = 1.2 × 10^−3^; *r*^*2*^ = 0.38).

The neighbor preference of the animals during the exploratory period correlated with the reward rate across the spatial alternation task (Figure 5A), justifying its inclusion in M3. The preference of the animals to have directional inertia and thereby perform large sweeps across the track impacts their performance during the first contingency (Figures 5B and 5C). However, even though directional inertia was prevalent for the animals during the exploratory period (Figure 1E), there was no significant correlation between the large sweep rate (sweeps greater than three arms) during the exploratory period and the total reward rate during the alternation task (Figure S5A; p = 0.4). This indicates that the sweeping affects the learning of the first contingencies but is not a prevalent part of the way the rats learn the subsequent contingencies.

The analyses above identified two factors that significantly correlate with the variability in learning spatial alternation: neighbor preferences displayed by the rats during the exploratory period of the task and the large sweep rate during the first alternation contingency, which reflects the learning rate of the rats. A critical goal of this study was to explicitly determine how to interpret variability in performance of spatial alternation behavior; therefore, we wanted to understand whether the variability in the behavior explained by these two factors was the same or different. We then asked whether the large sweep rate, and by extension the learning rate, captures a different aspect of the reward rate variability than that which is correlated with the neighbor transition frequency during the exploratory period (Figure 5A). We found that it does: the neighbor transition frequency during exploration did not correlate with the large sweep rate during the first alteration contingency (Figure S5B; p = 0.2).

In combination, the neighbor transition frequency during the exploratory period and the large sweep rate during the first alternation contingency account for 64.6% of the variance in the reward rates of the animals across the entire alternation task. We calculated the overall variance explained by fitting a multifactorial linear regression relating the transition frequency and large sweep rate to the overall reward rate during the alternation task. Consistent with the large sweep rate being correlated with the learning rate of the animals, the neighbor transition frequency during the exploratory period and the learning rate of the model (fit only to the second and third contingencies) account for 58.2% of the variance in the reward rates of the animals across the entire alternation task. The slight increase in variance captured by including the large sweep rate in the first contingency over including the learning rate of the model occurs because the large sweep rate in the first contingency is directly related to the amount of reward in the first contingency. If we compare the reward rate for contingencies 2–6, the neighbor transition frequency combined with the large sweep rate accounts for 64.9% of the variance, whereas the neighbor transition frequency combined with the learning rate accounts for 66.4% of the variance.

Therefore, we find that variability in spatial alternation reflects multiple factors, including the learning rates of the rats as well as their implementation of spatial preferences, such as a neighbor transition preference.

### Deviations of the behavior from the model point to generalization about the task

**Figure 6.**
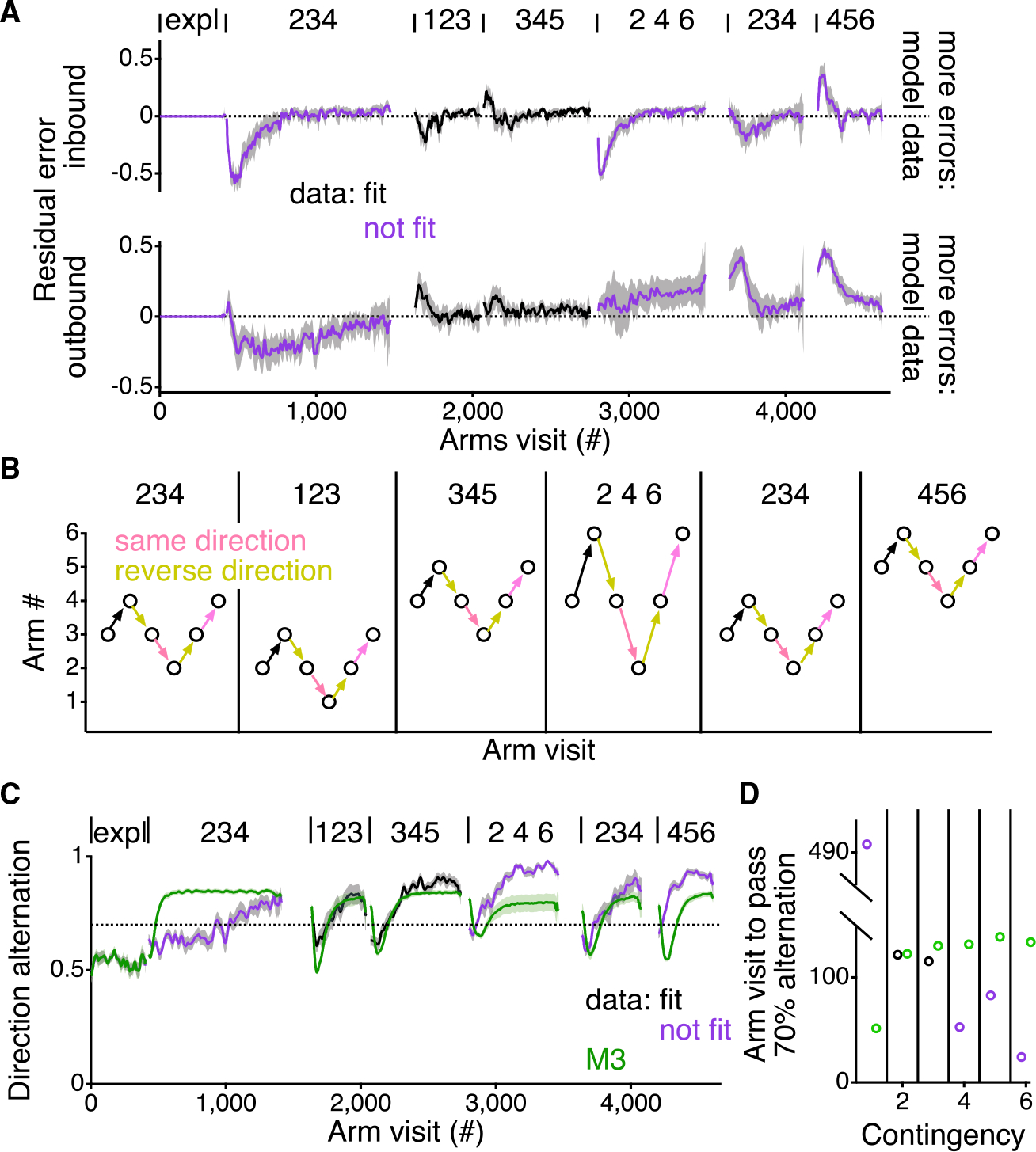
Model failure in later contingencies points to generalization of the task structure in the animals (A) Difference between the error likelihood for the rats and the model fit to the individual rats, averaged across all rats (±95% confidence interval around the mean). Positive residual values indicate that the model had higher error likelihoods, and negative residual values indicate that the model had lower error likelihoods. (B) Example sequence of correct arm visits for each contingency showing the common higher order structure shared across the contingencies. Pink arrows indicate continuing in the same direction, and yellow arrows indicate reversals in the direction of travel. All contingencies require alternation between continuing in the same direction and reversing direction. The first arm choice shown in each contingency is shown with a black arrow, as the prior direction is not defined. (C) Average (±SEM) rate of direction alternation in the animals and model M3 (green). Rate of direction alternation was calculated by first determining whether, on the current trial, the choice continued in the same direction as the previous trial or reversed direction from the previous trial and then that decision was compared with the previous trial, and if the current trial was the opposite direction choice than the previous trial, that trial was considered a direction alternation. The direction alternation for each animal was smoothed with a Gaussian filter with a SD of 10 trials. Vertical dotted line shows a direction alternation rate of 70%. (D) Arm visit number into each contingency where the mean of the animals or model M3 (green) increase in passing 70% direction alternation. For (A)–(C), contingencies are indicated as in Figure 1F. For (A) and (C), black shows the contingencies that were fit by the model and purple shows the contingencies that were not included in the fit.

Finally, we sought to understand systematic aspects of the learning that the model did not capture. Therefore, we evaluated the residuals of the model fitting by calculating the average difference between the inbound and outbound errors of the individual rats and the inbound and outbound errors of the model fits (Figure 6A). The residuals were minimal during the second and third contingencies, those contingencies used for fitting the model, indicating that, across the population of animals, the model did a good job of fitting these contingencies. As expected, the first contingency showed systematic deviations, with the model overall performing better than the animals.

Across the populations of animals, the outbound errors of the fourth contingency and inbound errors of the fifth and sixth contingencies were minimal. This demonstrates that the model fit to the second and third contingencies predicts the course of learning for these aspects of the behavior. This is particularly surprising for the outbound errors of the fourth contingency, since, in that contingency, the animals must learn to skip an arm to alternate between arms 2, 4, and 6. This good prediction of the model for these aspects of learning is partly what underlies the ability of the model to predict the ordering of the reward rate for the unfit contingencies (Figures 4C and 4D).

However, the residuals show that the model systematically does better than the animals for the inbound errors of the fourth contingency and systematically worse than the animals for the outbound errors of the fifth and sixth contingencies. One possible explanation for these differences would be that the animals are generalizing, something the model cannot do. Specifically, the animals could be learning about the higher order task structure in a manner that impairs performance in some cases and improves it in others.

We identified potential opportunities for generalization by noting that correct performance of each contingency involves alternating between same and opposite direction arm visits (Figure 6B). For example, in the first alternation contingency at arms 2, 3, and 4, a correct sequence of arm visits would be 3–4-3–2-3. The first two arm visits in this example—3–4—define the direction of travel as increasing in number. The next correct decision—3—reverses that direction to decrease in number. The correct decision after that—2—continues in decreasing in number, and the correct decision after that—3—reverses direction to increase in number.

We evaluated the evidence consistent with generalization by quantifying the frequency of direction alternation in the animals and the model across all six contingencies. We found that the animals are far slower than the model to increase their direction alternation in the first contingency, at least partly due to their continued sweeping from the exploratory period (Figure 5B). The levels of direction alternation of the model and the animals are more similar in the second and third contingencies, given that the model was fit to these contingencies. By contrast, the animals more rapidly reach higher rates of directional alternation and ultimately exhibit higher rates of directional alternation in contingencies 4, 5, and 6 as compared with the model (Figures 6C and 6D).

This increased amount of direction alternation by the animals is consistent with the pattern of error residuals that we see in the data (Figure 6A). The animals make fewer outbound errors than the model on the fifth and sixth contingencies because, once they find the center arm of the contingency, direction alternation would lead to appropriate transitions between the outer arms of the contingency. Increased direction alternation would also predict more inbound errors on the fourth contingency. Of the inbound errors that the animals make at the beginning of the fourth contingency (first 200 arm visits) 71.7% ± 1.9% of those errors are a part of a direction alternation sequence. That is the same fraction as the overall directional alternation of the animals during those trials (75.3% ± 2.1%; p = 0.24; two-tailed paired rank sum test). In comparison, the model has no capacity to learn about this higher order feature of the behavior and accordingly makes no inbound errors at the start of the fourth contingency (Figure 3A), since the center arm of the contingency, arm 4, does not change from the prior contingency.

## DISCUSSION

Can we rely on our intuition to understand the behavior of animals in complex tasks? We have shown that such an approach is misplaced when applied to spatial alternation behavior. With spatial alternation behavior, differences in learning rates have typically been interpreted as reflecting differences in the quality of each animal’s memory for past experiences (Fernandez-Ruiz et al., 2019; Jadhav et al., 2012; Kim and Frank, 2009; Mukai et al., 2019; Sigurdsson et al., 2010). We developed an automated six-arm spatial task and exposed rats to both an initial exploration period and a series of alteration contingencies, where the animal had to alternate among different subsets of arms (Figure 1). We then developed a series of RL models, first using memory alone and then, when that model proved insufficient, incorporating specific dynamic preferences that reflect favored arms or favored transitions between arms (Figure 2).

As we also found for the simpler, three-arm task (Kastner et al., 2020), the incorporation of these dynamic preferences was sufficient to produce a model that can learn the spatial alternation task as rapidly as the rats (Figures 2 and 3). The model that incorporated the dynamic preferences identified different learning parameters across animals and was able to predict aspects of individual animal behavior on data to which it had not been fit (Figures 4 and 6). The specific preferences added to fit the data included a neighbor transition preference that could be estimated from the initial exploration period. The strength of that preference for individual animals was highly predictive of the total amount of reward they received throughout the task (Figure 5A). Our results demonstrate that the dynamics of learning can be captured with relatively simple models that combine memory with dynamic preferences.

### Model successes and limitations point to continual learning and generalization

Animals seamlessly learn tasks over many different timescales, a characteristic difficult to reproduce in machine learning and artificial intelligence (Kirkpatrick et al., 2017; Zenke et al., 2017). Such continual learning can be precisely defined using our approach. Our model, fit to just two of the contingencies, predicts the behavior of the animals in other contingencies (Figure 6). That indicates that there need be no new type of learning for the fit and predicted contingencies, even though the specific application of the rule changes. However, in places where the model does not predict the behavior, it provides specific situations where the animal could change its learning and provide experimental substrate to better understand where and how animals continually learn.

The resulting description of how the animals might be learning the task is critical, as it provides another way to check assumptions that would be applied based off the rewards alone. The rats consistently learn the second alternation contingency faster than the first (Figure 2D). Just using the reward rate alone, one might have concluded that this faster learning reflects the rats utilizing generalized information about the structure of the task to enable that faster learning. Our results argue for a different interpretation. We find that the model-free M3 captures the reward rates of the rats on the second and third contingencies and that the slower learning of the first contingency is due, at least in part, to the persistence of the preference of the rats to perform large sweeps across the track (Figures 5B–5E). Thus, the application of the model, combined with detailed analyses of the starting conditions for learning allows us to account for what appears to be accelerated learning of the second alternation contingency without invoking generalization.

We do find evidence for generalization of learning at later contingencies, however. Specifically, the contingencies where the model less well predicts the behavior of the animals also allows for the generation of specific hypothesis as to what the animals might be doing. The model made more outbound errors in the final alternation contingencies and fewer inbound errors in the fourth contingency (Figure 6). We provide evidence that it is here that M3 is compromised by its inability to represent higher order structure that captures the pattern of directional movements (i.e., alternation between same and different direction choices). The rats show an overall increase in this direction alternation across contingencies, which serves them well in some cases (outbound trials in contingencies 5 and 6), but not in others (inbound trials in contingency 4). Beyond just providing evidence for the presence of generalization, when it occurs and how it manifests, this work provides an example of the value of having a rigorous and falsifiable framework, here in the form of model M3, to interpret animal behavior.

### Limitations of the study

In fitting the model, we employed an ABC-based fitting procedure. This uses an “on-policy” RL method, in which the model makes every choice for itself as it learns. It can be contrasted with an “off-policy,” imitation-based method, which, instead of attempting to match heuristically defined statistics of the behavior, maximizes the likelihood of each choice but based on prior choices made by the rats rather than the model itself. On-policy methods have previously been used to fit animal behavior (Lloyd et al., 2012; Luksys et al., 2009) and indeed are known to circumvent some critical problems with off-policy schemes (Kumar et al., 2019). Future work could concentrate on the trials after each change in contingency, when the animals and model must re-evaluate the causes of their new non-reinforcement to learn afresh. It would also be interesting to consider a wider array of contingences, including ones that are not symmetric, and to consider behavior in a larger track, where edge effects for the first and last arms may be less prominent.

## STAR★METHODS

### RESOURCE AVAILABILITY

#### Lead contact

Further information and requests for resources should be directed to and will be fulfilled by the lead contact, David Kastner (david.kastner2@ucsf.edu).

#### Materials availability

This study did not generate new unique reagents.

#### Data and code availability

All original code has been deposited at GitHub: https://github.com/dbkastner/sixArmWtrackModel and is publicly available as of the date of publication. All data has been deposited at GitHub: https://github.com/dbkastner/sixArmWtrackModel and is publicly available as of the date of publication. DOIs are listed in the Key Resources Table. Any additional information required to reanalyze the data reported in this paper will be made available upon reasonable request.

### EXPERIMENTAL MODEL AND SUBJECT DETAILS

All experiments were conducted in accordance with University of California San Francisco Institutional Animal Care and Use Committee and US National Institutes of Health guidelines. Rat datasets were collected from Long Evans rats, ordered from Charles River Laboratories, that were fed standard rat chow (LabDiet 5001). To motivate the rats to perform the task, reward was sweetened evaporated milk, and the rats were food restricted to ~85% of their basal body weight. Rats were singly housed during the experimentation. Age and sex of rats are indicated in Method Details below.

### METHOD DETAILS

#### Behavioral training and task

Two cohorts of rats, comprised of 6 males and 6 females each, were run on the automated behavior system. There were no systematic differences in reward probabilities between the male and female rats within the two cohorts (Figure S1C), so data from all animals were aggregated for subsequent analyses. The entire behavior took place over the course of 22 days for the first cohort and 21 days for the second cohort. The first cohort ran an extra day on the initial exploratory behavior, where the animals received rewards after visiting any arm of the track. At the start of the behavior the first cohort of rats were 4–5 months old, and the second cohort of rats were 3–4 months old.

The automated behavior system was custom designed and constructed out of acrylic. All parts of the behavior system were enclosed with walls. There were different symbols on each arm of the track serving as proximal cues, and there were distal cues distinguishing the different walls of the room. Pneumatic pistons (Clippard) opened and closed the doors. Python scripts, run through Trodes (SpikeGadgets), controlled the logic of the automated system. The reward wells contained an infrared beam adjacent to the reward spigot (Figure S1A). The automated system used the breakage of that infrared beam to progress through the logic of the behavior. In addition to the infrared beam and the spigot to deliver the reward, each reward well had an associated white light LED (Figure S1A).

Once animals were placed in the apparatus each day, no further experimenter contact was necessary until the end of the daily behavior. The apparatus contains four parts: 1) a six-armed track with reward wells at the end of each arm; 2) four rest boxes, each with a reward well; 3) corridors connecting the rest boxes to the track; and 4) doors to gate the pathway on and off the track for each rest box (Figure 1A). Each rat waits in its rest box until it is their turn to run on the track, at which point the doors open to enable entry for that rat onto the six-arm track. Once the rat gets onto the track, the door closes behind it, and it carries out its session on the six-arm track. At the end of the session, the doors back to the rest box open, and the rat returns to its rest box, and the next rats gains entry onto the track.

The sequence of operations of the track for the set of behaviors are: 1) the doors open to clear the path from a single rest box to the track. Concurrently, the lights in all reward wells on the track turn on (Figure S1A). 2) On the first break of a track reward well beam (Figure S1A) following the opening of the doors, the door to the track closes, thus starting the session of that animal. The animal then has a fixed maximum number of trials for its session, and the session ends when either that maximum has been reached or following a time limit of 30 min. Only one animal ever reached the time limit. 3) Upon breaking the beam at the reward well at the last trial of the session, all the reward well lights turn off, and the doors reopen to allow for passage back to the appropriate rest box. Concurrently, the light to the reward well in that rest box turns on. 4) Upon breaking the beam of the rest box reward well, the doors to the track close and the well delivers reward. The light of the rest box reward well turns off after reward delivery. 5) The doors to the track for the rest box for the next subject open, and the process repeats itself.

Each cohort of rats were divided into groups of four animals. The same groups were maintained throughout the duration of the experiment. Within a group, a given rat was always placed in the same rest box, and the four rats of a group serially performed the behavior. The rats had multiple sessions on the track each day. During the exploratory period of the behavior, the duration of a session was defined by a fixed number of rewards. The rats did 14–16 sessions (362–425 total trials) of exploration wherein the rats were rewarded at any arm visited if and only if it was not a repeat visit to the immediately preceding arm. This rule encouraged the rats to move around the track.

During the alternation task the duration of a session was defined either by a fixed number of center arm visits and at least one subsequent visit to any other arm, or a fixed amount of time on the track (30 min), whichever came first. During the alternation contingencies there were 3 sessions each day. For the first day of the 1^st^ alternation contingency there were 10 center arm visits per session, for the second day of the 1^st^ contingency and the first day of all other contingencies there were 20 center arm visits per session, and for all other days there were 40 center arm visits per session. Only one of the female rats reached the time limit, and it did so for only two sessions toward the beginning of the 1^st^ alternation contingency. For that one female we incorporated the trials that she ran on those sessions and did not distinguish the time out sessions for the analyses.

The algorithm underlying the spatial alternation task was such that three arms on the track had the potential for reward within a given contingency, for example during the contingency at arms 2–3-4, arms 2, 3, and 4 had the potential to be rewarded, and arms 1, 5, and 6 did not. Of those three arms we will refer to the middle of the three arms as the center arm (arm 3 in the above example) and the other two arms as the outer arms (arms 2 and 4 in the above example). Reward was delivered at the center arms if and only if: 1) the immediately preceding arm whose reward well infrared beam was broken was not the center arm. Reward was delivered at the outer two arms if and only if: 1) the immediately preceding arm whose reward well infrared beam was broken was the center arm, and 2) prior to breaking the infrared beam at the center arm, the most recently broken outer arm infrared beam was not the currently broken outer arm infrared beam. The one exception to the outer arm rules was at the beginning of a session, following the first infrared beam break at the center arm, where only the first condition had to be met if neither of the outer arms had yet to be visited.

For the running of the behavior, the infrared beam break determined an arm visit (Figure S1A); however, the rats would sometimes go down an arm, get very close to the reward wells, but not break the infrared beam. Therefore, for all the analyses described, an arm visit was defined as when a rat got close to a reward well. The times were extracted from a video recording of the behavior. These missed pokes were more frequent at the beginning of a contingency (Figure S1D), but overall were not that common. This proximity-based definition of an arm visit added additional arm visits to those defined by the infrared beam breaks, and by definition none of them could ever be rewarded, nor alter the logic of the underlying algorithm. However, because of the non-Markovian nature of the reward contingency, the missed pokes could affect the rewards provided for subsequent choices.

The different spatial alternation contingencies (Figure 1F) were chosen to present increasing challenges and multiple learning opportunities. The transition from the 1^st^ (2–3-4) to the 2^nd^ (1–2-3) contingency was designed to be relatively easy, since performing 2–3-4 would allow a rat to readily find the central arm of the new contingency. Finding this arm is critical to gaining consistent reward. The transition from the 2^nd^ (1–2-3) to the 3^rd^ (3–4-5) contingency was designed to be harder since the central arm (4) of the new contingency is not included in 1–2-3. The 4^th^ (2–4-6) contingency was designed to be the hardest since the animals have to skip an arm to get to the correct outer arm of the contingency. The 5^th^ (2–3-4) and 6^th^ (4–5-6) contingencies were chosen for comparison with the first three contingencies to understand the evolution of the ability of the animals to perform the task and generalize from previous experience.

As opposed to behaviors designed to study asymptotic performance, we did not use arbitrary criteria on a per animal basis for switching between the contingencies since the purpose of this task was to understand the continual learning and behavior of the rats. Furthermore, the automated system matched the number of inbound rewards of the animals, for all the animals that did not reach the time limit, ensuring that all animals had similar learning opportunities. We therefore switched to a new contingency the day after >80% of the animals received >80% reward over the course of a session. That ensured that by the time each contingency switched almost all the rats reached at least ~80% correct on a session during each contingency (Figure S1B).

### QUANTIFICATION AND STATISTICAL ANALYSIS

#### RL agents

For this behavior we chose a simplified output as the modeled feature: visiting arms. The nature of the algorithm that governs the behavior led to the choice of arm visits for the model, as arms visits are the only factor considered when evaluating rewards.

Given that each spatial alternation task could be framed as a partially observable Markov decision process, we adapted the working memory model of Todd et al. (2009) as the basis for our series of RL agents. The models specify rules governing propensities *m(a, s)* that contain the preferences of the agent of choosing arm a when the state is *s*. Models differ in terms of the various terms whose weighted sum defines the propensity.

In all agents (M1–3) the state is defined as the combination of the current arm location of the agent and the immediately preceding arm location of the agent, *s*_*t*_ = {*a*_*t*−*1*_,*a*_*t*_}. This is a simplification from the Todd et al. model, whereby *a*_*t*−*1*_ is always placed into the memory unit, effectively setting the gating parameter for the memory unit to always update the memory unit. Then, the first component of *m(a,s)* for all models is *b(a,s)*, which is a 6 × (36 + 6 + 1) matrix containing the transition contingencies to arm *a* from state *s*. The reason for the additional states beyond just the 6 × 6 arms by previous arms is to include the rest box in the possible locations to allow for the inclusion of the first arm visit of a session. In so doing that adds 6 + 1 additional states into the agents since the animal can be located in the rest box and can be located at any of the 6 arms having previously been in the rest box.

To provide the agents with additional spatial and transitional preferences we added components to the transition propensities. The first is an arm preference, *b*^*i*^*(a)* that is independent of the current state of the animal. The second is a preference for visiting arms that neighbor in space the current arm, $b^{n_{1}}\chi \left(a=a_{t}\pm 1\right)$, where χ() is the characteristic function that takes the value 1 if its argument is true (and ignoring arms outside the range 1 … 6) and *b*^*n*1^ is the (plastic) weight for this component. The third is a preference for visiting arms that are two removed, in space, from the current arm, $b^{n_{2}}\chi \left(a=a_{t}\pm 2\right)$. The neighbor arm preferences contain only single values, the preference to go to a neighboring arm, independent of the current arm location. The neighbor preferences were applied equally in both directions when possible (i.e., if the agent was at the end of the track the neighbor preference could only be applied to one direction).

To determine the probability of visiting each of the arms from a given state, the total propensity is passed through a softmax such that:

(Equation 1) $$p(a;s)=\frac{\exp(m(a,s))}{\sum_{b}\exp(m(b,s))}$$

The agent’s visit is then determined by a sample from this distribution. The choice of arm then determines the reward, *r*, which is either 0 or 1, based on the algorithm that governs the spatial alternation task. The probability of revisiting the current arm is set to zero, and the probabilities of going to the remaining arms sums to 1.

The model uses the REINFORCE policy gradient method (Williams, 1992) within the actor-critic framework of temporal difference learning, to update the propensities in the light of the presence or absence of reward. To do this, the agent maintains a state-long-run-value approximation, *V*(*s*), which functions as a lookup table, with one component for each state. The reward determines the state-value prediction error:

(Equation 2) $$\delta_{t}=r_{t}+\gamma V\left(s_{t+1}\right)-V\left(s_{t}\right)$$

where γ∈ [0,1) is a parameter of the model called the temporal discounting factor, which determines the contribution of future rewards to the current state.

𝛿_*t*_ is then used to update the preferences for all the components of the propensities and *V*(*s*). The state-based transition component is updated according to the rule:

(Equation 3) $$b(a,s)⇐b(a,s)(1-\omega )+\alpha \delta_{t}\times \left\{\begin{array}{l} 1-p(a;s),\;s=s_{t},\;a=a_{t}\\ -p(a;s),\;s=s_{t},\;a\neq a_{t}\\ 0,\;s\neq s_{t} \end{array}\right\}$$

where α ∈ [0,1] is a parameter of the model called the learning rate, which determines the amount by which all components of the propensities change based on the new information. *ω*∈ [0.001, 0.015] is also a parameter of the model called the forgetting rate, and determines how the propensities decay. The independent arm preference is updated according to the rule:

(Equation 4) $$b^{i}(a)⇐b^{i}(a)(1-\omega )+\alpha \delta_{t}\times \left\{\begin{array}{l} 1-p(a;s),\;a=a_{t}\\ -p(a;s),\;a\neq a_{t} \end{array}\right\}$$

The strength of the neighbor arm preferences is updated according to the rule:

(Equation 5) $$b^{i}(a)⇐b^{i}(a)(1-\omega )+\alpha \delta_{t}\times \left\{\begin{array}{l} 1-p\left(a=\left\{a_{t}+i,\;a_{t}-i\right\};\;s\right),\;a=a_{t}\pm i\\ -p\left(a=\left\{a_{t}+i,\;a_{t}-i\right\};\;s\right),\;a\neq a_{t}\pm i \end{array}\right\}$$

where *i* is either 1 or 2 depending on whether the propensity being calculated is the immediate neighbor preference or the 2 arm away preference. And, finally, the state-value approximation is updated according to the rule:

(Equation 6) $$V(s)⇐V(s)(1-\omega )+\alpha \delta_{t}\times \left\{\begin{array}{l} 1,\;s=s_{t}\\ 0,\;s\neq s_{t} \end{array}\right\}$$

The learning, α, and forgetting, *ω*, rates were the same for all the updating rules. This does not need to be the case, but since we found that a single learning and forgetting rate fit the data well, we did not feel there was a need to increase the complexity of the models by increasing the number of parameters.

#### Model fitting

The model was implemented in C++ and run and fit within Igor Pro (Wavemetrics). There were 7 arms at which the agent could be located, 6 track arms and 1 rest box “arm; ” whereas, there were only 6 arms to which the agent could transition. That means that the model implemented the transition from the rest box to the track but did not model the return to the rest box from the track, this was done so that all track arm visits during a session would be included in the analyses. For the working memory version of the model, there were, therefore, 43 states in which the agent could find itself. 36 states (6^2^) for all combinations for both the previous and current arm being one of the 6 track arms (6 of the states could never be visited since a return to the same arm is not allowed), an additional 6 states for the current arm being one of the 6 track arms and the previous “arm” being the rest box, and a final 1 state for the agent starting from the rest box.

We fit the various agents to individual animals by using an Approximate Bayesian Computation method. We found the parameters that minimized the average rms difference between the inbound and outbound errors of the individual animal and of the average of 200 different repeats of the model. The inbound and outbound fitting errors were summed with equal weighting to create the final fitting error. We used simulated annealing and ran the optimization at least 4 different times from different initial conditions. We chose the parameters with the minimal error. For each run of the model we used the same random number generating seed to minimize the random fluctuations between parameter sets (Daw, 2011).

We evaluated the error landscape of the fits to determine whether there were clear global minima for each animal. We found that there were indeed global minima that were distributed across the parameter space. Our fitting procedure reliably determined the vicinity of the global minima (see Figure S4 for an example), indicating that the differences among animals are interpretable and reflect differences in behavior.

#### Statistical methods

For testing violations from randomness of the population, we consider a random effects model. Let *θ* be the population probability of randomness. We construct a frequentist test of the null hypothesis that *θ* = 0.5 against the one-tailed alternative that *θ* < 0.5.

If we had *m* subjects we knew were random and *n* subjects we knew were not, with *m* + *n* = *N*, then the frequentist probability associated with the null hypothesis would depend on the tail probability of the fair binomial distribution for values as, or more extreme than *n*:

(Equation 7) $$p=\frac{1}{2^{N}}\sum_{k=0}^{n}\left(\begin{array}{l} N\\ K \end{array}\right)$$

In our case, we have subject *i* with probability *p*(*data*|*random* = *Φ*_*i*_. Thus, we have probabilities such as: $P(n=0|\mathrm{data;}\;\varphi )=\prod_{i=1}^{N}\Phi_{i},\;p(n=1|\mathrm{data};\;\varphi )=\prod_{i=1}^{N}\Phi_{i}\times \sum_{i=1}^{N}\frac{\left(1-\Phi_{i}\right)}{\Phi_{i}}$, etc. Thus, we have

(Equation 8) $$p=\sum_{j=0}^{N}p(n=j|\mathrm{data}\;;\varphi )\frac{1}{2^{N}}\sum_{k=0}^{n}\left(\begin{array}{l} N\\ K \end{array}\right)$$

In practice, we compute this by sampling *p*(*n* = *j*|*data; φ*. This makes the three p values for the different exploratory preferences of the rats: 1.08e-06, 6.31e-08 and 1.75e-06, respectively for the max arm probability, neighbor transition and directional inertia. Significant deviation from the random distribution was defined as 0.05/3 = 0.016667, the division by 3 was due to the Bonferroni correction for the 3 tests we ran for the different spatial preferences.

To determine whether M3 reaches 75% correct at rates more similar to the average performance of the rats than M2 (Figure 2D) we carried out a permutation test. We first generated 200 repeats of the performance of models M2 and M3, using the parameters for each model that maximized that model’s reward rate. We defined *d*3(true) and *d*2(true) as the average differences across contingencies 2–5 between the trial numbers where the average performance of M3 and M2 (respectively) pass 75% and the trial number when the average performance of the animals passed 75%. We then wrote Δ*d*(true) = *d*2(true-*d*3(true). We then randomly permuted the labels of the combined 400 repeats of M2 and M3, and created two notional M2 and M3 average curves, and Δ*d*(rep 1) Delta d(rep 1). We repeated this permutation 9999 more times. The permutation statistic is the quantile of Δ*d*(true) amongst the 10,000 Δ*d*(rep 1) … Δ*d*(rep 10,000).

## Supplementary Material

1

## Figures and Tables

**KEY RESOURCES TABLE:** 

REAGENT or RESOURCE	SOURCE	IDENTIFIER
Deposited data		
Data for this paper	This paper	https://github.com/dbkastner/sixArmWtrackModel
Experimental models: Organisms/strains		
Rattus norvegicus: Crl:LE strain code 006: Long Evans rats	Charles River Laboratories	RRID: RGD_2308852
Software and algorithms		
Code repository for this paper	This paper	https://github.com/dbkastner/sixArmWtrackModel

## Highlights

- Rats show specific spatial preferences in a six-arm track
- Spatial preferences expressed before task learning predict reward rate on task
- RL model captures multiple aspects of rat spatial alternation learning
- Rats generalize task structure later than expected from reward rate alone

## References

[R1] Anderson DJ, and Perona P (2014). Toward a science of computational ethology. Neuron 84, 18–31. 25277452 10.1016/j.neuron.2014.09.005

[R2] Awasthi A, Ramachandran B, Ahmed S, Benito E, Shinoda Y, Nitzan N, Heukamp A, Rannio S, Martens H, Barth J, (2019). Synaptotagmin-3 drives AMPA receptor endocytosis, depression of synapse strength, and forgetting. Science 363, eaav1483. 30545844 10.1126/science.aav1483

[R3] Brunton BW, Botvinick MM, and Brody CD (2013). Rats and humans can optimally accumulate evidence for decision-making. Science 340, 95–98. 23559254 10.1126/science.1233912

[R4] Daw ND (2011). Trial-by-trial data analysis using computational models. In Decision Making, Affect, and Learning: Attention and Performance XXIII, Delgado MR, Phelps EA, and Robbins TW, eds. (Oxford University Press), pp. 3–38.

[R5] Faraco G, Hochrainer K, Segarra SG, Schaeffer S, Santisteban MM, Menon A, Jiang H, Holtzman DM, Anrather J, and Iadecola C (2019). Dietary salt promotes cognitive impairment through tau phosphorylation. Nature 574, 686–690. 31645758 10.1038/s41586-019-1688-z PMC7380655

[R6] Fernandez-Ruiz A, Oliva A, Fermino de Oliveira E, Rocha-Almeida F, Tingley D, and Buzsáki G (2019). Long-duration hippocampal sharp wave ripples improve memory. Science 364, 1082–1086. 31197012 10.1126/science.aax0758 PMC6693581

[R7] Hochreiter S, and Schmidhuber J (1997). Long short-term memory. Neural Comput. 9, 1735–1780. 9377276 10.1162/neco.1997.9.8.1735

[R8] Jadhav SP, Kemere C, German PW, and Frank LM (2012). Awake hippocampal sharp-wave ripples support spatial memory. Science 336, 1454–1458. 22555434 10.1126/science.1217230 PMC4441285

[R9] Kastner DB, Gillespie AK, Dayan P, and Frank LM (2020). Memory alone does not account for the way rats learn a simple spatial alternation task. J. Neurosci. 40, 7311–7317. 32753514 10.1523/JNEUROSCI.0972-20.2020 PMC7534917

[R10] Kim SM, and Frank LM (2009). Hippocampal lesions impair rapid learning of a continuous spatial alternation task. PLoS One 4, e5494. 19424438 10.1371/journal.pone.0005494 PMC2674562

[R11] Kirkpatrick J, Pascanu R, Rabinowitz N, Veness J, Desjardins G, Rusu AA, Milan K, Quan J, Ramalho T, Grabska-Barwinska A, (2017). Overcoming catastrophic forgetting in neural networks. Proc. Natl. Acad. Sci. U S A 114, 3521–3526. 28292907 10.1073/pnas.1611835114 PMC5380101

[R12] Krakauer JW, Ghazanfar AA, Gomez-Marin A, MacIver MA, and Poeppel D (2017). Neuroscience needs behavior: correcting a reductionist bias. Neuron 93, 480–490. 28182904 10.1016/j.neuron.2016.12.041

[R13] Kumar A, Fu J, Tucker G, and Levine S (2019). Stabilizing off-policy Q-learning via bootstrapping error reduction. Preprint at arXiv. 10.48550/arXiv.1906.00949.

[R14] Li J, and Daw ND (2011). Signals in human striatum are appropriate for policy update rather than value prediction. J. Neurosci. 31, 5504–5511. 21471387 10.1523/JNEUROSCI.6316-10.2011 PMC3132551

[R15] Lintusaari J, Gutmann MU, Dutta R, Kaski S, and Corander J (2017). Fundamentals and recent developments in approximate bayesian computation. Syst. Biol. 66, e66–e82. 28175922 10.1093/sysbio/syw077 PMC5837704

[R16] Lloyd K, Becker N, Jones MW, and Bogacz R (2012). Learning to use working memory: a reinforcement learning gating model of rule acquisition in rats. Front. Comput. Neurosci. 6, 87. 23115551 10.3389/fncom.2012.00087 PMC3483721

[R17] Luksys G, Gerstner W, and Sandi C (2009). Stress, genotype and norepinephrine in the prediction of mouse behavior using reinforcement learning. Nat. Neurosci. 12, 1180–1186. 19684590 10.1038/nn.2374

[R18] Mukai J, Cannavó E, Crabtree GW, Sun Z, Diamantopoulou A, Thakur P, Chang C-Y, Cai Y, Lomvardas S, Takata A, (2019). Recapitulation and reversal of schizophrenia-related phenotypes in setd1a-deficient mice. Neuron 104, 471–487.e12. 31606247 10.1016/j.neuron.2019.09.014 PMC7010348

[R19] Poddar R, Kawai R, and Olveczky BP (2013). A fully automated high-throughput training system for rodents. PLoS One 8, e83171. 24349451 10.1371/journal.pone.0083171 PMC3857823

[R20] Ribeiro M, Brigas HC, Temido-Ferreira M, Pousinha PA, Regen T, Santa C, Coelho JE, Marques-Morgado I, Valente CA, Omenetti S, (2019). Meningeal gd T cell-derived IL-17 controls synaptic plasticity and short-term memory. Sci. Immunol. 4, eaay5199. 31604844 10.1126/sciimmunol.aay5199 PMC6894940

[R21] Rivalan M, Munawar H, Fuchs A, and Winter Y (2017). An automated, experimenter-free method for the standardised, operant cognitive testing of rats. PLoS One 12, e0169476. 28060883 10.1371/journal.pone.0169476 PMC5218494

[R22] Ruediger S, Vittori C, Bednarek E, Genoud C, Strata P, Sacchetti B, and Caroni P (2011). Learning-related feedforward inhibitory connectivity growth required for memory precision. Nature 473, 514–518. 21532590 10.1038/nature09946

[R23] Shin JD, Tang W, and Jadhav SP (2019). Dynamics of awake hippocampal-prefrontal replay for spatial learning and memory-guided decision making. Neuron 104, 1110–1125.e7. 31677957 10.1016/j.neuron.2019.09.012 PMC6923537

[R24] Sigurdsson T, Stark KL, Karayiorgou M, Gogos JA, and Gordon JA (2010). Impaired hippocampal-prefrontal synchrony in a genetic mouse model of schizophrenia. Nature 464, 763–767. 20360742 10.1038/nature08855 PMC2864584

[R25] Singer AC, and Frank LM (2009). Rewarded outcomes enhance reactivation of experience in the hippocampus. Neuron 64, 910–921. 20064396 10.1016/j.neuron.2009.11.016 PMC2807414

[R26] Singer AC, Karlsson MP, Nathe AR, Carr MF, and Frank LM (2010). Experience-dependent development of coordinated hippocampal spatial activity representing the similarity of related locations. J. Neurosci. 30, 11586–11604. 20810880 10.1523/JNEUROSCI.0926-10.2010 PMC2966971

[R27] Sorge RE, Martin LJ, Isbester KA, Sotocinal SG, Rosen S, Tuttle AH, Wieskopf JS, Acland EL, Dokova A, Kadoura B, (2014). Olfactory exposure to males, including men, causes stress and related analgesia in rodents. Nat. Methods 11, 629–632. 24776635 10.1038/nmeth.2935

[R28] Suri RE, and Schultz W (1999). A neural network model with dopamine-like reinforcement signal that learns a spatial delayed response task. Neuroscience 91, 871–890. 10391468 10.1016/s0306-4522(98)00697-6

[R29] Todd MT, Niv Y, and Cohen JD (2009). Learning to use working memory in partially observable environments through dopaminergic reinforcement. In Advances in Neural Information Processing Systems 21, Koller D, Schuurmans D, Bengio Y, and Bottou L, eds. (Curran Associates, Inc.), pp. 1689–1696.

[R30] Vasek MJ, Garber C, Dorsey D, Durrant DM, Bollman B, Soung A, Yu J, Perez-Torres C, Frouin A, Wilton DK, (2016). A complement-microglial axis drives synapse loss during virus-induced memory impairment. Nature 534, 538–543. 27337340 10.1038/nature18283 PMC5452615

[R31] Wang F, Ren S-Y, Chen J-F, Liu K, Li R-X, Li Z-F, Hu B, Niu J-Q, Xiao L, Chan JR, and Mei F (2020). Myelin degeneration and diminished myelin renewal contribute to age-related deficits in memory. Nat. Neurosci. 23, 481–486. 32042174 10.1038/s41593-020-0588-8 PMC7306053

[R32] Williams RJ (1992). Simple statistical gradient-following algorithms for connectionist reinforcement learning. Machine Learn. 8, 229–256.

[R33] Zenke F, Poole B, and Ganguli S (2017). Continual learning through synaptic intelligence. International Conference on Machine Learning, 3987–3995. PMC6944509 31909397

[R34] Zilli EA, and Hasselmo ME (2008). Modeling the role of working memory and episodic memory in behavioral tasks. Hippocampus 18, 193–209. 17979198 10.1002/hipo.20382 PMC2376903

