# Supplementary material for: Spatial preferences account for inter-animal variability during the continual learning of a dynamic cognitive task
Source: Cell Rep. Author manuscript; Available in PMC 2022 May 12. (PMC9096879; doi:10.1016/j.celrep.2022.110708)
Supplement: 1 [file NIHMS1799984-supplement-1.pdf]

Cell Reports, Volume *39*

Supplemental information

Spatial preferences account for inter-animal variability during the continual learning of a dynamic cognitive task

David B. Kastner, Eric A. Miller, Zhuonan Yang, Demetris K. Roumis, Daniel F. Liu, Loren M. Frank, and Peter Dayan

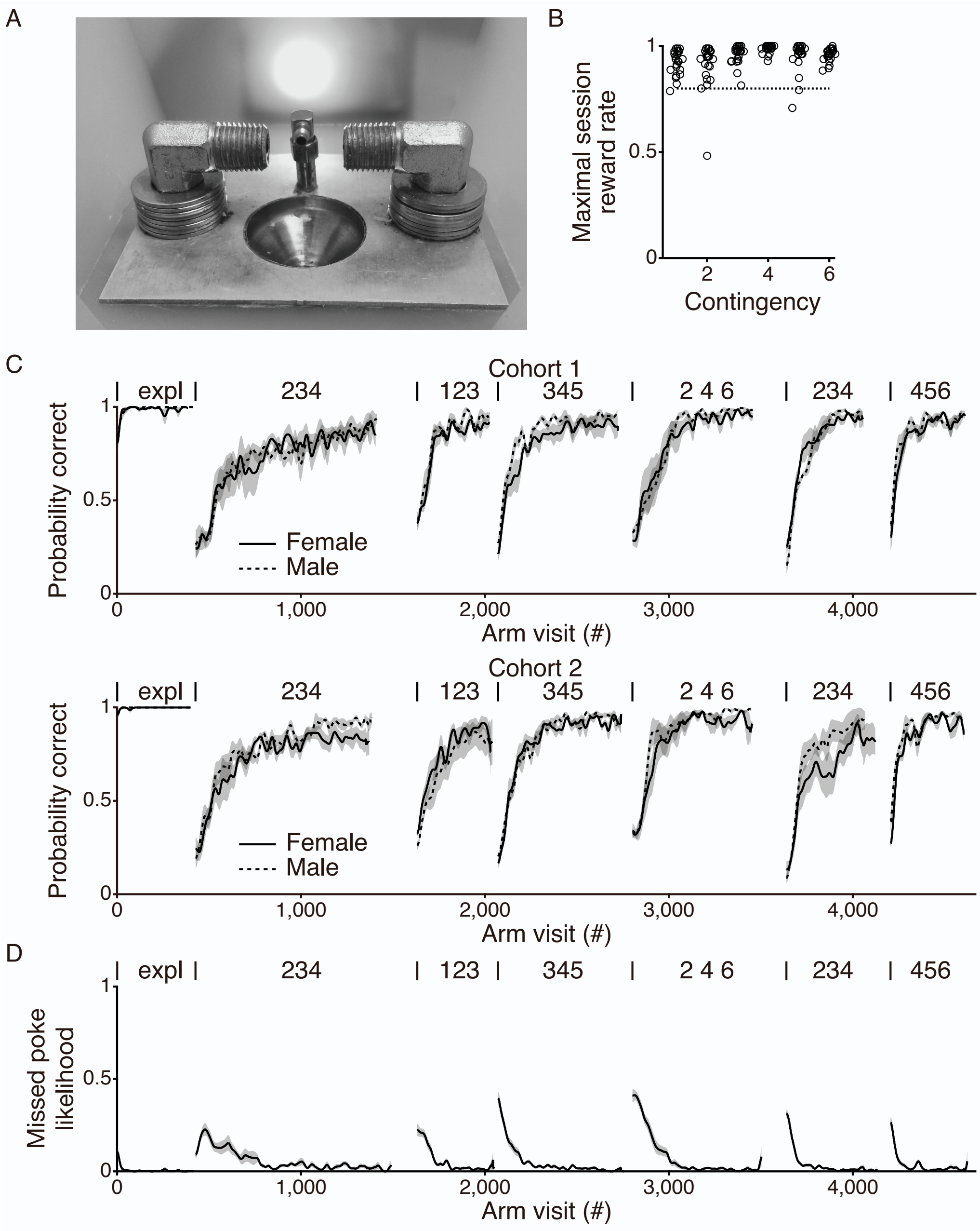

Figure S1. Males and females perform the behavior comparably across the two cohorts. Related to Figure 1. (A) Picture of a reward well showing the spigot through which milk is delivered, flanked by an IR LED and phototransistor, encased in metal elbows, to detect the position of the animal. Any unconsumed milk exits the track through the drain below the spigot. A light is illuminated directly behind the reward well when there is potential for reward delivery (see methods). Reward wells were made entirely out of metal. (B) Maximal reward rate in a session for each contingency and for all animals. Horizontal dotted line demarcates 80% correct. (C) Average probability of getting a reward for the male (dotted line) and female (solid line) rats in the first (top) and second (bottom) cohort. Within each contingency, curves smoothed with a Gaussian filter with a standard deviation of 10 arm visits and then averaged across the different animals. Thickness of the line indicates the sem. Contingencies indicated as in Fig 1F. (D) Average missed poke likelihood across all contingencies. Averaged across all rats. Thickness of line indicates sem. Contingencies indicated as in Fig 1F.

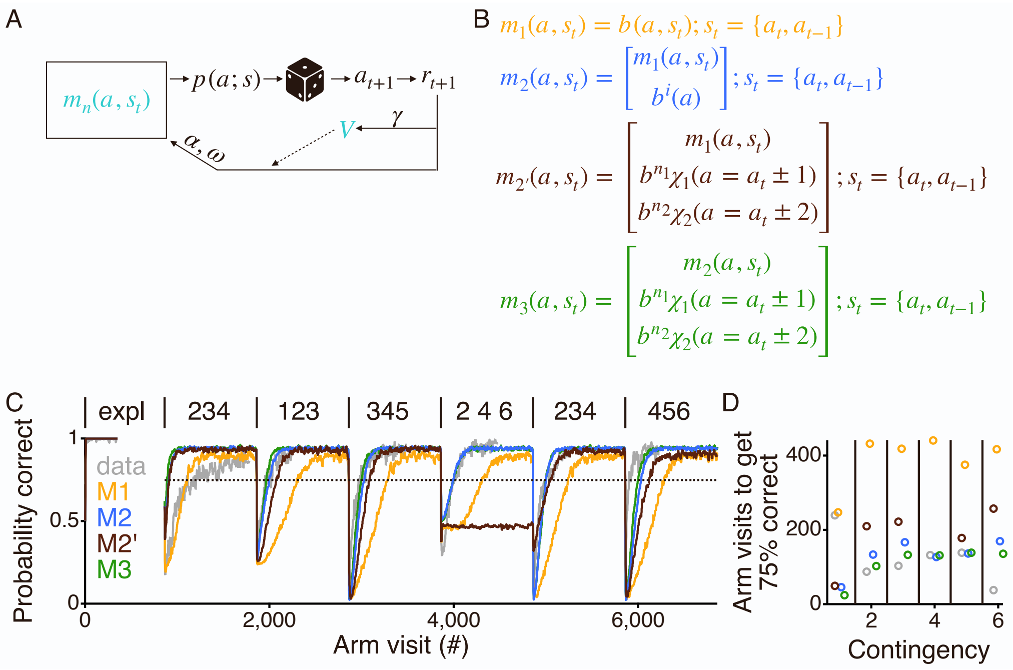

Figure S2. Comparison of additional model variant to data and other models. Related to Figure 2. (A) Graphic of RL agent. Colored symbols, $m_n(a, s_t)$ and V, indicate the components that change as the agent goes to arms, a, and does or does not get reward, r. (B) The different components of the propensities, $m_n(a, s_t)$, for the different models. The state of the agent, and therefore the probability of transitioning to each of the arms, $p(a; s)$, is defined by the current arm location, a_t, and the previous arm location, a_{t-1}, of the agent. $b^i(a)$ is the independent arm preference. $b^{n_1}\chi_1(a = a_t \pm 1)$ and $b^{n_2}\chi_2(a = a_t \pm 2)$ are the preference to transition to a neighbor 1 or 2 arms away, respectively. (C) Average reward probability of all animals (n = 24) across all contingencies (grey), and average behavior of 200 repeats of the models with parameters chosen to maximize the rewards received across all contingencies. The models were given extra arm visits to reach asymptotic behavior (after the endpoints of the grey curves for each contingency) to show more clearly the model's ability to learn the task. Dotted horizontal lines show 75% probability correct. Contingencies indicated as in Fig 1F. (D) Number of trials to pass 75% probability correct for the data (grey) and models. Colors refer to the different models from the previous panels. Brown points show the values for the model with state-based transition matrix, $b(a, s_t)$ and neighbor transition preferences, $b^{n_1}\chi_1(a = a_t \pm 1)$ and $b^{n_2}\chi_2(a = a_t \pm 2)$, without the independent arm preference, $b^i(a)$. There is no value for the fourth contingency, as the parameters that maximize total reward do not learn the fourth contingency well, even though there are other parameter sets that can learn the fourth contingency.

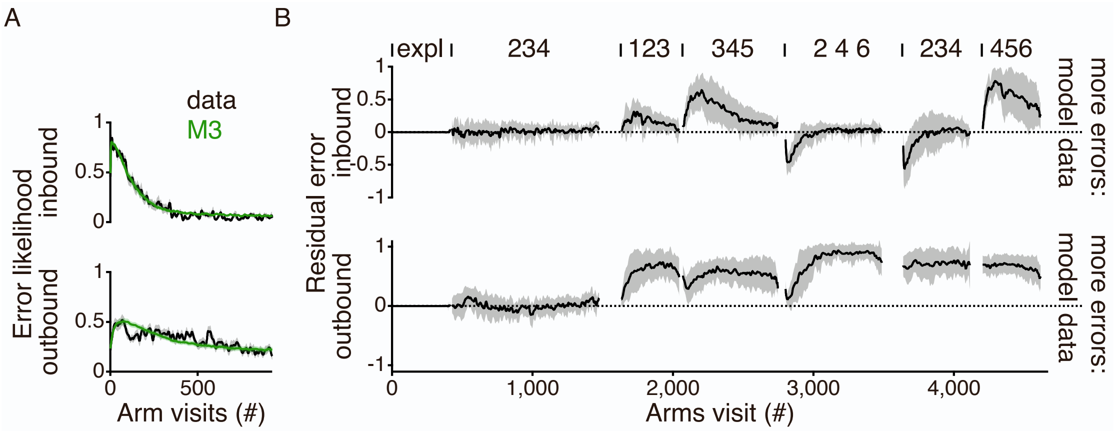

Figure S3. Fitting M3 to first contingency does not predict subsequent contingencies. Related to Figure 3. (A) Average inbound and outbound errors for the data (black) and model M3 (green) after fitting M3 to each individual animal. (B) Average residuals between the fit to each individual animal and the model fit only to the first alternation contingency. See Fig. 6A for comparison.

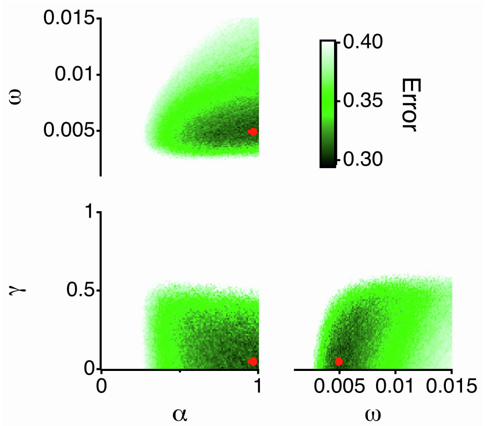

Figure S4. Error landscape for M3. Related to Figure 4. (A) Three-dimensional space of parameters projected onto the plane for the parameters from the fit. For instance, for the α/γ plot, the plane for the fit value of ω is chosen. The median and interquartile range for the parameters for the same rat from Fig. 3 for 24 fits are plotted as the red dot with errors bars in both axes (obscured by the dot). The color scale in the background shows the error between the model and the data.

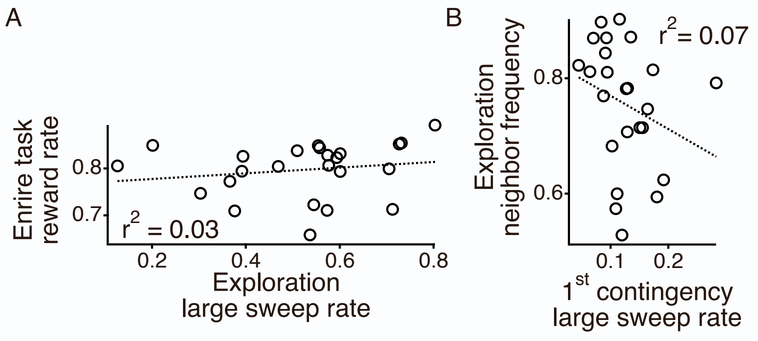

Figure S5. Lack of correlation between metrics. Related to Figure 5. (A) Entire task reward rate plotted relative to the large sweep (>3 arms) rate during the exploratory period. Dotted line shows linear fit. (B) Neighbor transition frequency during the exploratory period plotted relative to the large sweep rate during the first alternation contingency for each animal. Dotted line shows linear fit.
